# Supplementary material for: Aetiological and prognostic roles of frailty, multimorbidity and socioeconomic characteristics in the development of SARS-CoV-2 health outcomes: protocol for systematic reviews of population-based studies
Source: BMJ Open. 2022 Nov 22;12(11):e063573. doi: 10.1136/bmjopen-2022-063573 (PMC9684277; doi:10.1136/bmjopen-2022-063573)
Supplement: Author's manuscript [file bmjopen-2022-063573.draft_revisions.pdf]

BMJ Open is committed to open peer review. As part of this commitment we make the peer review history of every article we publish publicly available.

When an article is published we post the peer reviewers' comments and the authors' responses online. We also post the versions of the paper that were used during peer review. These are the versions that the peer review comments apply to.

The versions of the paper that follow are the versions that were submitted during the peer review process. They are not the versions of record or the final published versions. They should not be cited or distributed as the published version of this manuscript.

BMJ Open is an open access journal and the full, final, typeset and author-corrected version of record of the manuscript is available on our site with no access controls, subscription charges or pay-per-view fees (http://bmjopen.bmj.com).

If you have any questions on BMJ Open's open peer review process please email info.bmjopen@bmj.com

BMJ Open

Etiologic and prognostic roles of frailty, multimorbidity and socioeconomic characteristics in the development of SARS-CoV-2 infection and related severe health outcomes: protocol for systematic reviews of population-based studies

Journal:	*BMJ Open*
Manuscript ID	bmjopen-2022-063573
Article Type:	Protocol
Date Submitted by the Author:	06-Apr-2022

Complete List of Authors:	Makovski, Tatjana; Sante publique France,<br>Ghattas, Jinane; UCLouvain<br>Monnier Besnard, Stephanie; Sante publique France<br>Ambrozova, Monika; Institute of Health Information and Statistics of the Czech Republic<br>Vasinova, Barbora; Institute of Health Information and Statistics of the Czech Republic<br>Feteira-Santos , Rodrigo ; Universidade de Lisboa, Instituto de Saúde Ambiental, Faculdade de Medicina<br>Bezzegh, Peter; Government of Hungary<br>Ponce Bollmann, Felipe; Universidad Nacional de Educación a Distancia<br>Cottam, James; Sciensano<br>Haneef, Romana; Sante publique France<br>Devleesschauwer, Brecht; Sciensano, Epidemiology and public health<br>Speybroeck, Niko; Université catholique de Louvain, Institut de recherche santé et société (IRSS)<br>Nogueira, Paulo; Universidade de Lisboa Faculdade de Medicina<br>Forjaz, Maria João; National School of Public Health and Health Service Research Network for Chronic Diseases (Red de Investigación en Servicios de Salud en Enfermedades Crónicas/REDISSEC), Carlos III Institute of Health.<br>Coste, Joel; Sante publique France<br>Carcaillon-Bentata, Laure; Sante publique France

Keywords:	COVID-19, EPIDEMIOLOGY, PUBLIC HEALTH

SCHOLARONE™
Manuscripts

Etiologic and prognostic roles of frailty, multimorbidity and socioeconomic characteristics in the development of SARS-CoV-2 infection and related severe health outcomes: protocol for systematic reviews of population-based studies

Authors

Tatjana T. Makovski[1], Jinane Ghattas[2], Stéphanie Monnier-Besnard[1], Monika Ambrožová[3], Barbora Vašinová[3], Rodrigo Feteira-Santos[4,11], Peter Bezzegh[5], Felipe Ponce Bollmann[6], James Cottam[7,10], Romana Haneef[1], Brecht Devleesschauwer[7,9], Niko Speybroeck[2], Paulo Jorge Nogueira[4,11], Maria João Forjaz[8], Joël Coste[1], Laure Carcaillon-Bentata[1]

Authors' affiliations

[1] Public health France, France

[2] Institute of Health and Society, Université catholique de Louvain, Belgium

[3]Institute of Health Information and Statistics of the Czech Republic, Czech Republic

[4]Área Disciplinar Autónoma de Bioestatística, Faculdade de Medicina, Universidade de Lisboa, Lisboa, Portugal

[5]Directorate for Project Management, National Directorate General for Hospitals , Hungary

[6]Universidad Nacional de Educación a Distancia, Spain

[7]Department of Epidemiology and Public Health, Sciensano, Brussels, Belgium

[8]National Center of Epidemiology, Instituto de Salud Carlos III, REDISSEC and RICAPPS, Spain

[9]Department of Translational Physiology, Infectiology and Public Health, Ghent University, Merelbeke, Belgium

[10]Institute of Tropical Medicine, Antwerp, Belgium

[11]Instituto de Saúde Ambiental, Faculdade de Medicina, Universidade de Lisboa, Lisboa, Portugal

Corresponding author

Tatjana T. Makovski

Agence nationale de santé publique
12 rue du Val d'Osne
94415 Saint-Maurice Cedex France
www.santepubliquefrance.fr

tatjanamakovski@hotmail.com

For peer review only

Abstract

Introduction: There is growing evidence that the impact of COVID-19 crisis may be stronger for individuals with multimorbidity, frailty and lower socioeconomic status. Existing reviews focus on few, mainly short-term effects of COVID-19 and patients with single chronic disease. Information is also largely missing for population representative samples.

Applying population-based approach, the systematic reviews will have two objectives, to: 1) evaluate the etiologic roles of frailty, multimorbidity and socioeconomic status on SARS-CoV-2 infection probability, hospitalization, intensive care unit (ICU) admission, mechanical ventilation and COVID-19 related mortality among general population; 2) investigate the prognostic roles of frailty, multimorbidity and socioeconomic characteristics on the risk of hospitalization, ICU admission, mechanical ventilation, COVID-19 mortality, functioning, quality of life, disability, mental health and work absence.

Methods and analysis: For this ongoing work, four databases were searched: PubMed, Embase, WHO COVID-19 Global literature on coronavirus disease and PsycINFO, for the period between January 2020 and April 7 2021. Peer-reviewed published literature in English will be considered and all types of population-based studies. Studies using standard tools to assess multimorbidity such as disease count, comorbidity indices or disease combinations will be retained, as well as studies with standard scales and scores for frailty or measurement of a socioeconomic gradient. Initial search included 10,139 articles, 411 for full-text reading. Results will be summarized by risk factor, objective and outcome. The feasibility of meta-analysis will be determined by findings and will aim to better understand uncertainties of the results. Quality of studies will be assessed using standardized scales.

Ethics and dissemination: The study will be based on published evidence and it is exempt from ethical approval. This work is part of the Population Health Information Research Infrastructure (PHIRI) project. Dissemination of the results will imply conference presentation, submission for scientific publication and PHIRI project report.

Prospero registration: CRD42021249444.

For peer review only - http://bmjopen.bmj.com/site/about/guidelines.xhtml

Strengths and limitations of this study

- These systematic reviews will focus on exploring the etiologic and prognostic roles of multimorbidity, frailty and socioeconomic characteristics on short and long-term COVID-19 related health outcomes in population-based studies.
- Only peer-reviewed literature published in English and conducted on population representative samples will be eligible.
- The literature screening process, data extraction and study quality assessment will be performed by two reviewers independently.
- The findings will be summarized by risk factors, etiologic or prognostic role and study outcome; the findings will determine the feasibility of the meta-analysis.

For peer review only

For peer review only - http://bmjopen.bmj.com/site/about/guidelines.xhtml

Introduction

The early months of 2020 were marked with rapid growth in infections caused by the novel severe acute respiratory syndrome coronavirus 2 or SARS-CoV-2, leading to the state of a global pandemic declared by the World Health Organization (WHO) on March 11, 2020 (1). SARS-CoV-2 causes coronavirus disease 2019 (COVID-19) (2), which has led to severe health and health care management consequences around the world (3).

It has been demonstrated so far that age, male sex and comorbidity are the main risk factors for COVID-19 hospitalisations, intensive care unit (ICU) admissions and mortality (4-6). Specifically, infection fatality rates due to COVID-19 have been found to exponentially increase with age : 0.4% at age 55, 1.4% at age 65, 4.6% at age 75 and 15% at age 85 (7). In addition, having at least one comorbidity more than doubled the risk of hospitalisation while certain comorbidities, including severe kidney disease, diabetes, severe immunodeficiency or heart failure, among few others, increased this risk in particular, e.g., odds ratio (OR)/hazard ratio (HR) ≥ 3 for hospitalisation or death (8). Much remains to be clarified due to the lifespan of the new disease of only two years and the appearance of new virus variants (9) against which the same protection level conferred by existing vaccines is not guaranteed (10). In particular, regarding which and why certain population groups still display more severe symptoms and outcomes compared to others.

Frailty, as a marker of increased vulnerability due to accumulated deficits in multiple body systems (11) and multimorbidity, commonly defined as having two or more chronic conditions (12) are well recognized risk factors for adverse health outcomes in the elderly population, including hospitalisation, dependency and mortality (13-15). In the context of the COVID-19 pandemic, it is plausible that both frailty and multimorbidity increase the risk of SARS-CoV-2 infection and the development of severe outcomes by altering older people's biological background and immunity. Some studies have found that frailty and multimorbidity predicted COVID-19 patients' prognosis for hospitalisation, mortality and level of care at discharge (16-26). To date, however, most studies addressing these relationships are likely to be biased due to the inclusion of only severe COVID-19 cases admitted to the hospital. Whether the relationships of frailty and multimorbidity with COVID-19 infection and outcomes hold for non-severe forms of COVID-19, it is of major interest to improve risk stratification and public health guidance in the general population (27).

Genetic and biomedical factors are not the only determinants that may influence COVID-19 outcomes. During previous pandemics, lower socioeconomic status was associated with a higher disease burden. It is therefore important to consider social vulnerability (28). Several studies suggest that COVID-19, like other infectious diseases, disproportionally affects socially disadvantaged population groups and places them at a higher risk of disease severity. People of low socioeconomic status, ethnic minority groups (29) and crowded households (30) had higher risks of acquiring COVID-19 and higher hospitalisation rates. Overall, a large number of studies with very different methodological approaches (settings, type of adjustment) have been conducted to assess the role of socioeconomic factors in SARS-CoV-2 infection and outcomes. Therefore, it is important to synthesize the current knowledge from well-designed population-based studies in order to clarify and quantify the weight of each socioeconomic factor.

For peer review only

Finally, most of the investigated COVID-19 related health impacts appear so far to be of short-term, e.g. hospitalisations, ICU admissions or mortality. Evidence on long-term effects such as potential mental health difficulties, impact on quality of life or functioning, among others, in COVID-19 patients is limited (31, 32), but it will certainly grow as the pandemic prolongs.

The above assumptions justify the relevance, need and importance of summarizing current knowledge using a systematic review approach regarding the etiologic and prognostic roles of frailty, multimorbidity and socioeconomic characteristics in developing COVID-19 and related short and long-term health outcomes, among general population. The Prospero registry for systematic reviews (33) was cross-checked to ensure the novelty of the research questions.

Objectives

This paper aims to describe the methodology, which will be used for conducting systematic reviews intended at synthesising knowledge on the roles of frailty, multimorbidity and socioeconomic characteristics in the acquisition and evolution of COVID-19 related health outcomes. Two main study objectives have been determined.

The first objective is to explore the effects of frailty, multimorbidity and socioeconomic characteristics on SARS-CoV-2 infection risk in the general population. The goal is to consider the general population at risk of developing SARS-CoV-2 infection or related outcomes and to estimate a potential increase in risk associated with a given risk factor (frailty or multimorbidity or socioeconomic characteristics). The outcomes of interest are infection by SARS-CoV-2, hospitalisation for COVID-19, ICU admission, mechanical ventilation and mortality due to COVID-19.

The second objective is to explore the prognostic effects of frailty, multimorbidity and socioeconomic characteristics in SARS-CoV-2 infected subjects from the general population. The goal is to consider SARS-CoV-2 infected individuals from the general population and estimate a potential increase in risk of developing severe short- and long-term outcomes associated with a given risk factor (frailty, multimorbidity or socioeconomic characteristics). The outcomes of interest are death due to COVID-19, hospitalization, ICU admission, mechanical ventilation, functioning, quality of life, disability, mental health difficulties and work absence/sick leave.

Methods and analysis

Protocol and registration

This protocol is written along the requirements of Preferred Reporting Items for Systematic Review and Meta-Analysis Protocols (PRISMA-P) 2015 checklist (34). The study is registered in the Prospero registry for systematic review protocols under the registration number: CRD42021249444.

Should there be any substantial amendments to this protocol in the course of the study, they will be fully documented and included in dissemination, such as reports and scientific publications.

Eligibility criteria

We applied the Population, Exposure, Comparator and Outcomes (PECO) framework (35) to clearly specify research objectives. This framework has been increasingly recognized in population health, environmental and

For peer review only

occupational health fields due to the importance of identifying the exposure and clarifying its association with the outcome; (35, 36). The study objectives are hence further detailed as follows:

Objective 1

P Population = general or well-defined population SARS-CoV-2 negative

E Exposure = (either) frailty, multimorbidity, lower socioeconomic status

C Comparator = general population with no frailty or no multimorbidity or with better socioeconomic status

O Outcome(s) = infection by SARS-COV-2, hospitalization for COVID-19, ICU admission, mechanical ventilation, mortality due to COVID-19.

Objective 2

P Population = general or well-defined population SARS-CoV-2 infected (COVID-19 diagnosed, with e.g. polymerase chain reaction (PCR) test, medical imaging or similar)

E Exposure = (either) frailty, multimorbidity, lower socioeconomic status

C Comparator = SARS-CoV-2 infected subjects from the population with no frailty or no multimorbidity or with better socioeconomic status

O Outcome(s) = death due to COVID-19, hospitalization for COVID-19, ICU admission, mechanical ventilation, , functioning, quality of life, disability, mental health difficulties and work absence/sick leave.

In the absence of a widely agreed definition of multimorbidity (37, 38), studies with any valid measurement of multimorbidity, such as disease count (≥ 2, ≥ 3 diseases or similar), comorbidity/multimorbidity indices or disease combinations, will be considered. Similarly, only studies with well-established frailty scales and scores will be retained. Likewise, literature looking at the role of the socioeconomic gradient in COVID-19 related outcomes will be included. All age groups will be taken into account considering that socioeconomic differences may be observed across all ages and that multimorbidity is increasingly present among younger individuals as well as among older adults (39).

Eligible studies and settings

To identify population based studies, the following definitions will apply: “a population-based study is defined as a study of properties of a well-defined population, such as individuals residing in a defined geographic region in a given time period”(40) and “population-based studies aim to answer research questions for defined populations; answers should be generalizable to the whole population addressed in the study hypothesis, not only to the individuals included in the study” (41). Therefore, the following examples of population-based studies may be eligible for our reviews: community-dwelling, hospital-based, nursing homes, homes for elderly, or similar, as long as the population in the study is representative of the country or region from which the sample was drawn.

Study designs

All types of etiologic and prognostic observational studies will be eligible, such as cohorts, cross-sectional studies, case-control studies or ecological studies (for socioeconomic determinants only). Interventions and clinical trials will be excluded. Only published peer-reviewed original studies written in English will be considered.

For peer review only - http://bmjopen.bmj.com/site/about/guidelines.xhtml

For peer review only

Information sources and search strategy

The following databases were be explored: PubMed, Embase, WHO COVID-19 Global literature on coronavirus disease (42) and PsycINFO. The search strategy considers all possible variations of the terms: multimorbidity, frailty, terms defining socioeconomic characteristics, COVID-19 and study design types. The search strategy was adjusted to the technical specificities of each database. The example of the search strategy in PubMed is presented in Table 1. The search strategy for remaining three databases is presented in Annex 1.

Table 1 PubMed search strategy

#1	Covid-19 (Adjusted from Lazarus et al.)(43)	((((((((((((((((((("Betacoronavirus"[MeSH Terms] OR "Coronavirus Infections"[MeSH Terms]) OR "COVID-19"[Supplementary Concept]) OR "Coronavirus"[MeSH Terms]) OR "Severe Acute Respiratory Syndrome Coronavirus 2"[Supplementary Concept]) OR "2019nCoV"[All Fields]) OR "betacoronavirus*"[All Fields]) OR "corona virus*"[All Fields]) OR "coronavirus*"[All Fields]) OR "coronovirus*"[All Fields]) OR "CoV"[All Fields]) OR "CoV2"[All Fields]) OR "COVID"[All Fields]) OR (("COVID-19"[Supplementary Concept] OR "COVID-19"[All Fields]) OR "covid19"[All Fields])) OR ((((((("COVID-19"[All Fields] OR "covid 2019"[All Fields]) OR "Severe Acute Respiratory Syndrome Coronavirus 2"[Supplementary Concept]) OR "Severe Acute Respiratory Syndrome Coronavirus 2"[All Fields]) OR "2019 ncov"[All Fields]) OR "SARS CoV 2"[All Fields]) OR "2019nCoV"[All Fields]) OR (("wuhan"[All Fields] AND ("Coronavirus"[MeSH Terms] OR "Coronavirus"[All Fields])) AND (2019/12/1:2019/12/31[Date - Publication] OR 2020/1/1:2020/12/31[Date - Publication])))) OR "HCoV-19"[All Fields]) OR "nCoV"[All Fields]) OR "SARS CoV 2"[All Fields]) OR "SARS2"[All Fields]) OR "SARSCoV"[All Fields]) OR (((("sars virus"[MeSH Terms] OR ("sars"[All Fields] AND "virus"[All Fields])) OR "sars virus"[All Fields]) OR ("sars"[All Fields] AND "CoV"[All Fields])) OR "sars cov"[All Fields])) OR (("Severe Acute Respiratory Syndrome Coronavirus 2"[Supplementary Concept] OR "Severe Acute Respiratory Syndrome Coronavirus 2"[All Fields]) OR "SARS CoV 2"[All Fields])) OR "severe acute respiratory syndrome cov*"[All Fields]) AND (2019/11/17:3000/12/31[Date - Entry] OR 2019/11/17:3000/12/31[Date - Publication]) OR "COVID-19"[MeSH Terms] OR "SARS-Cov-2"[MeSH Terms] OR "SARS CoV-2" OR "SARS-CoV-2" OR SARSCoV2 OR "CoV-2" OR "covid 19" OR covid2019 OR "covid-2019" OR "novel CoV" OR "corona pandemic*" OR "wuhan virus*" OR "CoV 2" OR ((wuhan OR hubei OR huanan) AND ("severe acute respiratory" OR pneumonia*) AND outbreak*)
#2	Frailty	frailty OR frail OR frailty[MeSH Terms]
#3	Multimorbidity (Adjusted from Makovski et al.)(44)	multimorbidity OR "multi-morbidity" OR "multi morbidity" OR multimorbidities OR "multi-morbidities" OR "multi morbidities" OR multimorbid OR "multi-morbid" OR "multi morbid" OR comorbidity OR "co-morbidity" OR "co morbidity" OR comorbidities OR "co-morbidities" OR "co morbidities" OR comorbid OR "co morbid" OR "multiple chronic conditions" OR "multiple chronic diseases" OR "multiple conditions" OR "multiple diseases" OR "multiple disorders" OR polymorbid* OR "poly-morbid*" OR "poly morbid*" OR polypath* OR pluripath* OR multipath* OR "multi path*" OR "multi-path*" OR "multiple pathologies" OR "disease cluster" OR "disease clusters" OR "disease pattern" OR "disease patterns" OR "concurrent chronic diseases" OR "multiple chronic disorders" OR multimorbidity[MeSH Terms] OR comorbidity[MeSH Terms]

For peer review only - http://bmjopen.bmj.com/site/about/guidelines.xhtml

#4	Socioeconomic characteristics	"socio economic" OR "socio-economic" OR socioeconomics OR "socio-economics" OR "socio economics" OR socioeconomic OR "social difference" OR "social differences" OR "social inequality" OR "social inequalities" OR "socioeconomic inequality" OR "socioeconomic inequalities" OR "social disparity" OR "social disparities" OR education OR literacy OR "socioprofessional" OR "socio-professional" OR "socio professional" OR "social conditions" OR "social class" OR "social classes" OR "social class"[MeSH Terms] OR "socioeconomic factors"[MeSH Terms] OR health status disparities[MeSH Terms] OR income OR poverty OR deprivation OR rural OR urban OR "housing deprivation" OR homeless OR houseless OR homelessness OR ethnic* OR race OR emigrant OR immigrant OR migrant OR "minority group" OR "minority groups" OR disadvantaged OR "marital status" OR ((characteristics OR factors OR status) AND (economic OR social OR educational)) OR ((composition OR characteristics OR size) AND (family OR household))
#5	Study design	"cross-sectional" OR "cross sectional" OR "case-control" OR "case control" OR cohort OR longitudinal OR "ecological study" OR "ecological studies" OR "ecological design" OR "ecological designs" OR observational OR "observational study" OR "observational studies" OR "observational design" OR "observational designs" OR "prospective study" OR "prospective studies" OR "prospective design" OR "prospective designs" OR "retrospective study" OR "retrospective studies" OR "retrospective design" OR "retrospective designs" OR "prospective observational study" OR "prospective observational studies" OR "retrospective observational study" OR "retrospective observational studies" OR case-control studies[MeSH Terms] OR cohort studies[MeSH Terms] OR cross-sectional studies[MeSH Terms]
#6		#2 OR #3 OR #4
#7		#1 AND #5 AND #6

The search was restricted in each database using date (January 2020 to April 7, 2021) and language (English) filters. Reference lists of retained studies will be explored for potentially omitted relevant records. For studies where pertinent data are not presented but seem to be available, study authors will be contacted for details. If deemed relevant, update of the literature search will be performed.

Study records

Data management

The literature will be managed using Rayyan (45), EndNote and Excel. Records retrieved from the four databases will be exported in EndNote for deduplication. Title and abstract screening will be performed in Rayyan, a web and mobile app for systematic reviews. Full-text articles will be read in EndNote and data will be extracted in customised Excel tables. Two researchers will perform the screening process and data extraction. Considering the actuality of the topic, the quantity of the literature envisaged and the need for timely information, several researchers will be involved in the screening process. For example, two researchers will assume the role of the first reviewer while several colleagues will act as the second reviewer. Weekly meetings will ensure a clear understanding of the study process. In addition, prior to the start of the study selection, a pilot test is foreseen; for instance, homogeneity in the understanding of the exclusion and inclusion criteria among reviewers will be compared and explained on a minimum of 50 studies.

For peer review only

For peer review only - http://bmjopen.bmj.com/site/about/guidelines.xhtml

Selection process

Applying the exclusion criteria displayed in List 1, two reviewers will independently screen all records by title and abstract (records screening phase) to select studies for full-text reading (reports screening phase) (46).

List 1: Reasons for exclusion from the systematic review (title/abstract or records screening phase)

1 = Language other than English
2 = Not an original research (e.g. editorial, protocol, etc.) or no original results
3 = Unrelated topic
4 = Not a population-based study
5 = Subpopulation (medical staff, students, pregnant women, etc.)
6 = Duplicate

The criteria above will be used in a hierarchical order to reduce the discrepancy in selected exclusion criteria between the reviewers, implying that should there be more than one criterion applicable to a study, the one higher on the list will be assigned.

Exclusion criteria in List 2 will apply for selecting studies during a full-text (reports) reading phase.

List 2: Reasons for exclusion from the systematic review (full-text reading or reports screening phase)

1 = Not a population-based study
2 = SARS-CoV-2 infection diagnosis not clear
3 = Study does not consider people with frailty or multimorbidity OR does not contain information on socioeconomic characteristics
4 = Outcome not within the scope of our objectives
5 = Outcome measurement tool not clear
6 = Risk factor measurement tool not clear
7 = Subpopulation (e.g. pregnant women, health care workers, students etc.)
8 = Not an original research (e.g. editorial, protocol, review, conference abstract, grey literature, etc.), no original results or not peer-reviewed
9 = The same or largely the same population already considered in another study for the same outcome
10 = Clinical trial or intervention study
11 = Qualitative study
12 = Descriptive study and/or no measure of the association of interest
13 = Other (explain)

The records will be rated as eligible/not eligible/questionable (47) at both screening stages. Any disagreement between the reviewers will be resolved by consensus in referral to this protocol; a third reviewer will be consulted if necessary. Disagreement between the reviewers will be reported in percentages (48).

For peer review only - http://bmjopen.bmj.com/site/about/guidelines.xhtml

All articles will be sorted during the screening process by type of exposure factors, e.g., frailty and multimorbidity on one side (as biomedical determinants) and socioeconomic characteristics on the other, and by objective (objective 1 or 2).

Retained studies will be kept for further analyses.

Data items

Six extraction tables for each exposure and objective will be prepared. They will include: study title, authors, year of publication, study design, study setting and duration, population, sample size, COVID-19 diagnostic criteria, risk factors and study outcomes and means by which they were assessed, as well as the measurement of the association between variables of interest, and study adjustment factors.

Two reviewers will extract data independently and compare for agreement (48).

Risk of bias in individual studies

Two reviewers will separately assess a risk of bias for each study retained for final reporting. Any disagreement will be resolved by consensus; otherwise, a third reviewer will be consulted. The Newcastle-Ottawa scales will be used to assess the quality of case-control and cohort studies (49). Each of these scales contains eight items grouped in three categories: selection, comparability and outcome or exposure (for cohort and case-control studies, respectively). Selection items refer to e.g. representativeness of the sample, ascertainment of the exposure, definitions of case and control group, etc. Comparability items inquire on comparability between e.g. cases and controls or between cohorts based on the study design or analysis. Outcomes category items account for the assessment of the outcome or adequacy of the follow up, while exposure items account for e.g. ascertainment of the exposure or response rate. Each scale item can carry a maximum of one point, except for the comparability group, where up to two points for an item can be assigned, totalling a maximum of nine points for study quality. Higher score indicates higher study quality.

An adjusted version of the Newcastle-Ottawa scale will be used to assess the quality of cross-sectional studies (50, 51). Here maximum of 10 points can be assigned.

Abovementioned scales will also apply to assess study quality of ecological studies (for socioeconomic risk factor only), depending of the ecological study design.

Data synthesis

The literature review is ongoing. The search included 10,139 articles in title and abstract screening phase, of which 411 were retained for full-text reading. The screening process results will be presented with a PRISMA diagram (52). Given the interest in several risk factors, the PRISMA diagram may be adjusted, as suggested in Fig. 1.

Fig. 1 PRISMA 2020 flow diagram for new systematic reviews, which included searches of databases and registers only (adjusted)

**BM = Biomedical, SE= Socioeconomic*

For peer review only

The final set of retained studies will be described using standardised tables. They will be summarised within each objective, by type of risk factor and study outcome and presented in tabulated form. Study characteristics will be described as well as their main findings.

When possible, meta-analysis models will be performed by each objective, for each risk factor and for a specific outcome with the aim to better understand the uncertainties (random error and bias) of the obtained results. The feasibility of performing meta-analyses will depend on additional criteria, such as number of studies applying the same measurement tools for risk factors and evaluating the same outcomes, as well as reporting odds ratio, hazard ratio, risk ratio or slope. For the meta-analysis, a minimum of four studies will be required. In studies assessing the role of socioeconomic inequalities on health outcomes, unadjusted studies stratified by age group will be sought to avoid overadjustment (53). For studies assessing the roles of biomedical factors, adjusted analyses will be preferred. Adjustment factors will be thoroughly reported. Random effects meta-analyses will be performed with R software using the metafor package (54). Findings will be presented with forest plot(s). Publication bias will be visually assessed using funnel plots and formally tested using Begg and Mazumdar's rank correlation test (55) and Egger's regression test (56). When significant publication bias will be present, Duval and Tweedie's trim-and-fill method will be used to explore the impact on model estimates (57). Heterogeneity will be explored by computing I^2 statistic (58). When feasible, sensitivity analysis or meta-regression using study quality as a covariate will be conducted. Other covariates such as proper operationalisation of multimorbidity or frailty (e.g. number and list of diseases clearly provided or proper definition used), mean age or gender proportion, may also be considered.

Patient and public involvement

Patients and/or the public were not involved in the design of the protocol and will not be involved in conducting the study, reporting or dissemination plans of the research.

Discussion

This protocol aims to present the methodology that will be applied to systematically assess the evidence on the etiologic and prognostic roles of multimorbidity, frailty and socioeconomic risk factors in the development of SARS-CoV-2 infection and COVID-19 related severe short- and long-term health outcomes, among the general population. These reviews should inform on the quantity and quality of the relevant literature and summarise the existing evidence. In addition, the reviews will report which were the preferred assessment methods for the risk factors and most frequent study outcomes considered, as well as which were the statistical approaches and adjustment factors across studies.

To date, no systematic review on the link between multimorbidity and SARS-CoV-2 infection and outcomes has been identified. Though not specifically focusing on patients with multimorbidity, Kuswardhani et al. (59) have summarised evidence on the association between the Charlson’s score and poor short-term outcomes in COVID-19 patients. This score is still frequently used to assess health status in patients with multiple conditions, in the absence of a more specific and widely accepted multimorbidity tool. We plan to elaborate on other

For peer review only

multimorbidity measurements and broader COVID-19 related consequences, and investigate the possible etiologic role of multimorbidity in the illness acquisition and development. In the context of ageing societies where an increasing number of people live with multiple conditions, scarcity of evidence and public health advice for this population group in the COVID-19 crisis is unacceptable. It is essential to inform how to best evaluate the risks for people with multiple number and combinations of diseases to provide clinicians and policy makers with adequate advice for the most optimal and evidence-based decisions (27).

On the other hand, several systematic reviews have been conducted among the frail population (22, 25). However, these focus on hospital settings, usually single centre or few multicentre settings and short-term outcomes; prognostic roles of frailty are also mainly explored. None of the identified reviews investigated the role of frailty in the development of COVID-19 related outcomes in population representative samples. Our systematic review intends to build on the existing evidence by examining the occurrence of a wide number of COVID-19 health outcomes accounting for etiologic as well as prognostic roles of frailty, among the general population.

Similarly, reviews show existing disparities in COVID-19 outcomes in lower socioeconomic groups and ethnic minorities. Specific contexts such as low level of education, poor housing conditions and crowded households could play a role in worsening COVID-19 outcomes (60-62). As above, no systematic review to date has investigated COVID-19 outcomes and severity related to socioeconomic gradient in well-defined population based studies. We, therefore aim to fill this gap. Whereas the three types of factors (multimorbidity, frailty and socioeconomic factors) will be considered and analyzed separately, the similar use of a population-based approach justify the definition of a common methodology and the sharing of the first steps of the reviews performed.

A potential limitation of the reviews is that only publications written in English will be considered. With this, we possibly risk to overlook national population representative studies, which may have been in the necessity of the crisis as well as for local purposes, released in local languages exclusively. On the other hand, a publication in peer-reviewed international journals in English may also be regarded as an additional study quality check. A strength and a limitation could be the number of scientists who are planned to be involved in the study. This may accelerate the review process, which is desired when information is crucial. However, one needs to consider the heterogeneity among reviewers in understanding the study method and the review criteria. We will overcome this by conducting a pilot test and holding regular team and individual meetings with involved parties. Moreover, the two first reviewers, experts in respectful areas, will remain the same throughout the screening and data extraction process. Accurately following validated guidelines in preparing this protocol, conducting and reporting the study will help to limit any possible biases to a minimum.

We are hopeful that these reviews will provide relevant and timely novel evidence to facilitate public health decisions in the midst of the COVID-19 crisis and identify current gaps in the literature to direct further research.

For peer review only

Ethics and dissemination

The study will be based on published evidence and is exempt from ethical approval. The dissemination will include submission for publication in a peer-reviewed journal related to the field and presentation at a scientific conference. In addition, a PHIRI project report will be written.

Acknowledgements

We wish to thank Population Health Information Research Infrastructure (PHIRI) coordination team for initiating and financing this project. We thank documentation experts from Santé Publique France, Laetitia Haroutunian and Sandra Kerzanet, for their inputs in drafting this protocol.

For peer review only

Authors' contributions

LCB, JC, BD, PJN and MJF identified the research questions. All authors were involved in the conceptualisation of the search strategy. The final strategy was designed in collaboration with the documentation experts (LH, SK), who assisted in deciding on appropriate databases, keywords and search optimization.

TM and JG will share the role of a first reviewer, each reviewing half of the retrieved articles; the role of a second reviewer will be divided between LCB, SMB, RH, RFS, FPB, JC, MA, BV, PB, PJN. Acting first and the second reviewer will independently evaluate retrieved records for eligibility and inclusion in the systematic reviews and they will independently extract relevant data. The third-party (JC), if necessary, will resolve any disagreement between the two reviewers.

TM, JG, JC, BD and LCB formulated the first version of this article. All authors revised and approved the final version of the article.

Funding statement

These systematic reviews are part of Population Health Information Research Infrastructure (PHIRI) project (63). PHIRI is funded by the European Union's Horizon 2020 research and innovation program under grant agreement No: 101018317.

The protocol was developed in collaboration with all listed institutions. The funding body was not involved in the design of the study; it will not be involved in the collection, analysis and interpretation of data or in writing the manuscript. The content of this publication will represent the views of the authors only and not of the European Commission or any other body of the European Union. The European Commission does not take any responsibility for use that may be made of the information it will contain.

Competing interests statement

Authors declare no competing interests.

For peer review only - http://bmjopen.bmj.com/site/about/guidelines.xhtml

For peer review only

For peer review only - http://bmjopen.bmj.com/site/about/guidelines.xhtml

REFERENCES

1. Cucinotta D, Vanelli M. WHO Declares COVID-19 a Pandemic. Acta Biomed. 2020;91(1):157-60.
2. Novel Coronavirus (2019-nCoV) Situation Report – 22 (World Health Organization). 2020 [Available from: https://www.who.int/docs/default-source/coronaviruse/situation-reports/20200211-sitrep-22-ncov.pdf.
3. World Health Organisation (WHO). [Available from: https://www.who.int/emergencies/diseases/novel-coronavirus-2019/situation-reports.
4. Cheng Y, Luo R, Wang K, Zhang M, Wang Z, Dong L, et al. Kidney disease is associated with in-hospital death of patients with COVID-19. Kidney Int. 2020;97(5):829-38.
5. Kumar A, Arora A, Sharma P, Anikhindi SA, Bansal N, Singla V, et al. Is diabetes mellitus associated with mortality and severity of COVID-19? A meta-analysis. Diabetes Metab Syndr. 2020;14(4):535-45.
6. Grasselli G, Greco M, Zanella A, Albano G, Antonelli M, Bellani G, et al. Risk Factors Associated With Mortality Among Patients With COVID-19 in Intensive Care Units in Lombardy, Italy. JAMA Intern Med. 2020;180(10):1345-55.
7. Levin AT, Hanage WP, Owusu-Boaitey N, Cochran KB, Walsh SP, Meyerowitz-Katz G. Assessing the age specificity of infection fatality rates for COVID-19: systematic review, meta-analysis, and public policy implications. European Journal of Epidemiology. 2020;35(12):1123-38.
8. Himmels JPW, Borge TC, Brurberg KG, Gravningen KM. COVID-19: COVID-19 and risk factors for hospital admission, severe disease and death Oslo: Norwegian Institute of Public Health; 2021.
9. Johns Hopkins University of Medicine; Coronavirus resource centre. COVID-19 Dashboard by the Center for Systems Science and Engineering (CSSE) at Johns Hopkins University (JHU) [Available from: https://coronavirus.jhu.edu/map.html.
10. Andrews N, Stowe J, Kirsebom F, Toffa S, Rickeard T, Gallagher E, et al. Effectiveness of COVID-19 vaccines against the Omicron (B.1.1.529) variant of concern. medRxiv. 2021:2021.12.14.21267615.
11. Clegg A, Young J, Iliffe S, Rikkert MO, Rockwood K. Frailty in elderly people. Lancet. 2013;381(9868):752-62.
12. van den Akker M, Buntinx F, Knottnerus JA. Comorbidity or multimorbidity: what's in a name? A review of literature. European Journal of General Practice. 1996;2(2):65-70.
13. Fried LP, Tangen CM, Walston J, Newman AB, Hirsch C, Gottdiener J, et al. Frailty in older adults: evidence for a phenotype. J Gerontol A Biol Sci Med Sci. 2001;56(3):M146-56.
14. Rockwood K, Song X, MacKnight C, Bergman H, Hogan DB, McDowell I, et al. A global clinical measure of fitness and frailty in elderly people. Cmaj. 2005;173(5):489-95.
15. Coste J, Valderas JM, Carcaillon-Bentata L. Estimating and characterizing the burden of multimorbidity in the community: A comprehensive multistep analysis of two large nationwide representative surveys in France. PLoS Med. 2021;18(4):e1003584.
16. Iaccarino G, Grassi G, Borghi C, Ferri C, Salvetti M, Volpe M. Age and Multimorbidity Predict Death Among COVID-19 Patients: Results of the SARS-RAS Study of the Italian Society of Hypertension. Hypertension. 2020;76(2):366-72.
17. McQueenie R, Foster HME, Jani BD, Katikireddi SV, Sattar N, Pell JP, et al. Multimorbidity, polypharmacy, and COVID-19 infection within the UK Biobank cohort. PLoS One. 2020;15(8):e0238091.
18. Woolford SJ, D'Angelo S, Curtis EM, Parsons CM, Ward KA, Dennison EM, et al. COVID-19 and associations with frailty and multimorbidity: a prospective analysis of UK Biobank participants. Aging Clin Exp Res. 2020;32(9):1897-905.
19. Zhang XM, Jiao J, Cao J, Huo XP, Zhu C, Wu XJ, et al. Frailty as a predictor of mortality among patients with COVID-19: a systematic review and meta-analysis. BMC Geriatr. 2021;21(1):186.
20. Yang Y, Luo K, Jiang Y, Yu Q, Huang X, Wang J, et al. The Impact of Frailty on COVID-19 Outcomes: A Systematic Review and Meta-analysis of 16 Cohort Studies. J Nutr Health Aging. 2021;25(5):702-9.
21. Saragih ID, Advani S, Saragih IS, Suarilah I, Susanto I, Lin CJ. Frailty as a mortality predictor in older adults with COVID-19: A systematic review and meta-analysis of cohort studies. Geriatr Nurs. 2021;42(5):983-92.
22. Pranata R, Henrina J, Lim MA, Lawrensia S, Yonas E, Vania R, et al. Clinical frailty scale and mortality in COVID-19: A systematic review and dose-response meta-analysis. Arch Gerontol Geriatr. 2021;93:104324.
23. Maltese G, Corsonello A, Di Rosa M, Soraci L, Vitale C, Corica F, et al. Frailty and COVID-19: A Systematic Scoping Review. J Clin Med. 2020;9(7).
24. Kastora S, Kounidas G, Perrott S, Carter B, Hewitt J, Myint PK. Clinical frailty scale as a point of care prognostic indicator of mortality in COVID-19: a systematic review and meta-analysis. EClinicalMedicine. 2021;36:100896.

For peer review only

25. Cosco TD, Best J, Davis D, Bryden D, Arkill S, van Oppen J, et al. What is the relationship between validated frailty scores and mortality for adults with COVID-19 in acute hospital care? A systematic review. Age Ageing. 2021;50(3):608-16.
26. Dumitrascu F, Branje KE, Hladkowicz ES, Lalu M, McIsaac DI. Association of frailty with outcomes in individuals with COVID-19: A living review and meta-analysis. Journal of the American Geriatrics Society. 2021;69(9):2419-29.
27. Mair FS, Foster HM, Nicholl BI. Multimorbidity and the COVID-19 pandemic - An urgent call to action. J Comorb. 2020;10:2235042x20961676.
28. Mamelund S-E, Shelley-Egan C, Rogeberg O. The association between socioeconomic status and pandemic influenza: Systematic review and meta-analysis. PloS one. 2021;16(9):e0244346.
29. Niedzwiedz CL, O'Donnell CA, Jani BD, Demou E, Ho FK, Celis-Morales C, et al. Ethnic and socioeconomic differences in SARS-CoV-2 infection: prospective cohort study using UK Biobank. BMC medicine. 2020;18:1-14.
30. Nash D, Qasmieh S, Robertson M, Rane M, Zimba R, Kulkarni S, et al. Household factors and the risk of severe COVID-like illness early in the US pandemic. medRxiv. 2020.
31. Himmels JPW, Qureshi SA, Brurberg KG, Gravningen KM. COVID-19: Long-Term Effects of COVID-19. Rapid review. Oslo: Norwegian Institute of Public Health, 2021. 2021.
32. Dumitrascu F, Branje KE, Hladkowicz ES, Lalu M, McIsaac DI. Association of frailty with outcomes in individuals with COVID-19: A living review and meta-analysis. J Am Geriatr Soc. 2021;69(9):2419-29.
33. National Institute for Health Research. PROSPERO; International prospective register of systematic reviews [Available from: https://www.crd.york.ac.uk/PROSPERO/.
34. Shamseer L, Moher D, Clarke M, Ghersi D, Liberati A, Petticrew M, et al. Preferred reporting items for systematic review and meta-analysis protocols (PRISMA-P) 2015: elaboration and explanation. Bmj. 2015;350:g7647.
35. Morgan RL, Whaley P, Thayer KA, Schünemann HJ. Identifying the PECO: A framework for formulating good questions to explore the association of environmental and other exposures with health outcomes. Environ Int. 2018;121(Pt 1):1027-31.
36. Morgan RL, Thayer KA, Bero L, Bruce N, Falck-Ytter Y, Ghersi D, et al. GRADE: Assessing the quality of evidence in environmental and occupational health. Environ Int. 2016;92-93:611-6.
37. Almirall J, Fortin M. The coexistence of terms to describe the presence of multiple concurrent diseases. J Comorb. 2013;3:4-9.
38. Johnston MC, Crilly M, Black C, Prescott GJ, Mercer SW. Defining and measuring multimorbidity: a systematic review of systematic reviews. Eur J Public Health. 2019;29(1):182-9.
39. St Sauver JL, Boyd CM, Grossardt BR, Bobo WV, Rutten LJF, Roger VL, et al. Risk of developing multimorbidity across all ages in an historical cohort study: differences by sex and ethnicity. BMJ open. 2015;5(2):e006413.
40. Gail MH. Population based study. In: Armitage, &, Colton, editors. Encyclopedia of biostatistics: Wiley, Chichester 1998. p. 3450.
41. Lieb R. Population-Based Study. In: Gellman MD, Turner JR, editors. Encyclopedia of Behavioral Medicine. New York, NY: Springer New York; 2013. p. 1507-8.
42. (WHO) WHO. COVID-19 Global literature on coronavirus disease 2022 [Available from: https://search.bvsalud.org/global-literature-on-novel-coronavirus-2019-ncov/.
43. Lazarus JV, Palayew A, Rasmussen LN, Andersen TH, Nicholson J, Norgaard O. Searching PubMed to Retrieve Publications on the COVID-19 Pandemic: Comparative Analysis of Search Strings. J Med Internet Res. 2020;22(11):e23449.
44. Makovski TT, Schmitz S, Zeegers MP, Stranges S, van den Akker M. Multimorbidity and quality of life: Systematic literature review and meta-analysis. Ageing Res Rev. 2019;53:100903.
45. Ouzzani M, Hammady H, Fedorowicz Z, Elmagarmid A. Rayyan-a web and mobile app for systematic reviews. Syst Rev. 2016;5(1):210.
46. Page MJ, Moher D, Bossuyt PM, Boutron I, Hoffmann TC, Mulrow CD, et al. PRISMA 2020 explanation and elaboration: updated guidance and exemplars for reporting systematic reviews. Bmj. 2021;372:n160.
47. Alderson P, Green S, Higgins JPT, editors. Cochrane Reviewers' Handbook 4.2.2 [updated March 2004]. In: The Cochrane Library, Issue 1, Chichester, UK: John Wiley & Sons, Ltd. 2004. .
48. Centre for Reviews and Dissemination [CRD]. Systematic Reviews: CRD's Guidance for Undertaking Reviews in Healthcare. 3rd edn. . CRD University of York, York Publishing Services Ltd, York, . 2009.
49. Wells GA, Shea B, O'Connell D, Peterson J, Welch V, Losos M, et al. The Newcastle-Ottawa Scale (NOS) for assessing the quality if nonrandomized studies in meta-analyses. Available from: http://www.ohri.ca/programs/clinical_epidemiology/oxford.asp [

For peer review only

50. Herzog R, Álvarez-Pasquin MJ, Díaz C, Del Barrio JL, Estrada JM, Gil Á. Are healthcare workers' intentions to vaccinate related to their knowledge, beliefs and attitudes? a systematic review. BMC Public Health. 2013;13(1):154.
51. Modesti PA, Reboldi G, Cappuccio FP, Agyemang C, Remuzzi G, Rapi S, et al. Panethnic differences in blood pressure in Europe: a systematic review and meta-analysis. PloS one. 2016;11(1):e0147601.
52. Page MJ MJ, Bossuyt PM, Boutron I, Hoffmann TC, Mulrow CD, et al. . The PRISMA 2020 statement: an updated guideline for reporting systematic reviews. . BMJ 2021:372:n71. .
53. Schisterman EF, Cole SR, Platt RW. Overadjustment bias and unnecessary adjustment in epidemiologic studies. Epidemiology. 2009;20(4):488-95.
54. Viechtbauer W. Conducting Meta-Analyses in R with the metafor Package. Journal of Statistical Software. 2010;36(3):1 - 48.
55. Begg CB, Mazumdar M. Operating Characteristics of a Rank Correlation Test for Publication Bias. Biometrics. 1994;50(4):1088-101.
56. Egger M, Davey Smith G, Schneider M, Minder C. Bias in meta-analysis detected by a simple, graphical test. Bmj. 1997;315(7109):629-34.
57. Duval S, Tweedie R. Trim and fill: A simple funnel-plot-based method of testing and adjusting for publication bias in meta-analysis. Biometrics. 2000;56(2):455-63.
58. Higgins JP, Thompson SG. Quantifying heterogeneity in a meta-analysis. Stat Med. 2002;21(11):1539-58.
59. Tuty Kuswardhani RA, Henrina J, Pranata R, Anthonius Lim M, Lawrensia S, Suastika K. Charlson comorbidity index and a composite of poor outcomes in COVID-19 patients: A systematic review and meta-analysis. Diabetes & Metabolic Syndrome: Clinical Research & Reviews. 2020;14(6):2103-9.
60. Vist GE, Arentz-Hansen EH, Vedøy TF, Spilker RACS, Hafstad EV, Giske L. Incidence and severe outcomes from COVID-19 among immigrant and minority ethnic groups and among groups of different socio-economic status: A systematic review. 2021.
61. Wachtler B, Michalski N, Nowossadeck E, Diercke M, Wahrendorf M, Santos-Hövener C, et al. Socioeconomic inequalities and COVID-19–A review of the current international literature. 2020.
62. Khanijahani A, Iezadi S, Gholipour K, Azami-Aghdash S, Naghibi D. A systematic review of racial/ethnic and socioeconomic disparities in COVID-19. International journal for equity in health. 2021;20(1):1-30.
63. European Commission: Horizon 2020. Population Health Information Research Infrastructure [Available from: https://cordis.europa.eu/project/id/101018317/fr.

For peer review only

For peer review only - http://bmjopen.bmj.com/site/about/guidelines.xhtml

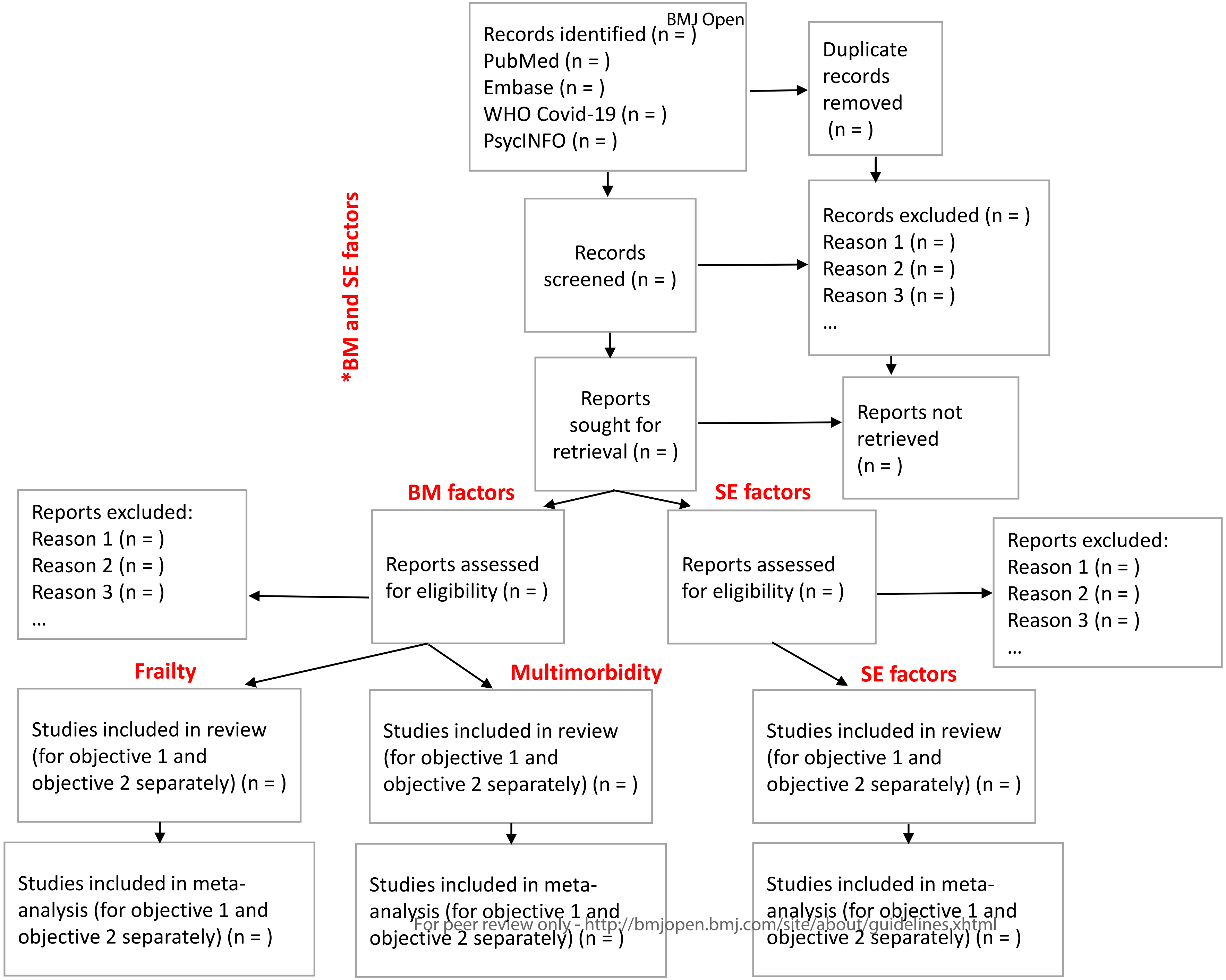

For peer review only - http://bmjopen.bmj.com/site/about/guidelines.xhtml

WHO COVID-19 Global literature on coronavirus disease strategy

((tw:("socio economic" OR "socio-economic" OR socioeconomics OR "socio-economics" OR "socio economics" OR socioeconomic OR "social difference" OR "social differences" OR "social inequality" OR "social inequalities" OR "socioeconomic inequality" OR "socioeconomic inequalities" OR "social disparity" OR "social disparities" OR education OR literacy OR "socioprofessional" OR "socio-professional" OR "socio professional" OR "social conditions" OR "social class" OR "social classes" OR "health status disparity" OR "health status disparities" OR income OR poverty OR deprivation OR rural OR urban OR "housing deprivation" OR homeless OR houseless OR homelessness OR ethnic$ OR race OR emigrant OR immigrant OR migrant OR "minority group" OR "minority groups" OR disadvantaged OR "marital status" OR ((characteristics OR factors OR status) AND (economic OR social OR educational)) OR ((composition OR characteristics OR size) AND (family OR household)))) OR (tw:(multimorbidity OR "multi-morbidity" OR "multi morbidity" OR multimorbidities OR "multi-morbidities" OR "multi morbidities" OR multimorbid OR "multi-morbid" OR "multi morbid" OR comorbidity OR "co-morbidity" OR "co morbidity" OR comorbidities OR "co-morbidities" OR "co morbidities" OR comorbid OR "co morbid" OR "multiple chronic conditions" OR "multiple chronic diseases" OR "multiple conditions" OR "multiple diseases" OR "multiple disorders" OR polymorbid$ OR "poly-morbidity" OR "poly-morbidities" OR "poly morbidity" OR "poly morbidities" OR polypath$ OR pluripath$ OR multipath$ OR "multi pathology" OR "multi pathologies" OR "multi-pathology" OR "multi-pathologies" OR "multiple pathologies" OR "disease cluster" OR "disease clusters" OR "disease pattern" OR "disease patterns" OR "concurrent chronic diseases" OR "multiple chronic disorders")) OR (tw:(frailty OR frail))) AND tw:("cross-sectional" OR "cross sectional" OR "case-control" OR "case control" OR cohort OR longitudinal OR "ecological study" OR "ecological studies" OR "ecological design" OR "ecological designs" OR observational OR "observational study" OR "observational studies" OR "observational design" OR "observational designs" OR "prospective study" OR "prospective studies" OR "prospective design" OR "prospective designs" OR "retrospective study" OR "retrospective studies" OR "retrospective design" OR "retrospective designs" OR "prospective observational study" OR "prospective observational studies" OR "retrospective observational study" OR "retrospective observational studies")

For peer review only

PsycINFO strategy

S1

"cross-sectional" OR "cross sectional" OR "case-control" OR "case control" OR cohort OR longitudinal OR "ecological study" OR "ecological studies" OR "ecological design" OR "ecological designs" OR observational OR "observational study" OR "observational studies" OR "observational design" OR "observational designs" OR "prospective study" OR "prospective studies" OR "prospective design" OR "prospective designs" OR "retrospective study" OR "retrospective studies" OR "retrospective design" OR "retrospective designs" OR "prospective observational study" OR "prospective observational studies" OR "retrospective observational study" OR "retrospective observational studies" OR DE("Longitudinal Studies" OR "Followup studies" OR "Retrospective studies" OR "Cohort analysis")

S2

"socio economic" OR "socio-economic" OR socioeconomics OR "socio-economics" OR "socio economics" OR socioeconomic OR "social difference" OR "social differences" OR "social inequality" OR "social inequalities" OR "socioeconomic inequality" OR "socioeconomic inequalities" OR "social disparity" OR "social disparities" OR education OR literacy OR "socioprofessional" OR "socio-professional" OR "socio professional" OR "social conditions" OR "social class" OR "social classes" OR "health status disparity" OR "health status disparities" OR income OR poverty OR deprivation OR rural OR urban OR "housing deprivation" OR homeless OR houseless OR homelessness OR ethnic* OR race OR emigrant OR immigrant OR migrant OR "minority group" OR "minority groups" OR disadvantaged OR "marital status" OR ((characteristics OR factors OR status) AND (economic OR social OR educational)) OR ((composition OR characteristics OR size) AND (family OR household)) OR DE ("Disadvantaged" OR "Economic Disadvantage" OR "Economic Inequality" OR "Economic Resources" OR "Family Socioeconomic Level" OR "Income (Economic)" OR "Lower Class" OR "Poverty" OR "Rural Health" OR "Socioeconomic Status" OR "Socioeconomic Factors" OR "Social Class" OR "Urban Health" OR "Racial and Ethnic Differences" OR "Racial Disparities" OR "Health Disparities")

S3

multimorbidity OR "multi-morbidity" OR "multi morbidity" OR multimorbidities OR "multi-morbidities" OR "multi morbidities" OR multimorbid OR "multi-morbid" OR "multi morbid" OR comorbidity OR "co-morbidity" OR "co morbidity" OR comorbidities OR "co-morbidities" OR "co morbidities" OR comorbid OR "co morbid" OR "multiple chronic conditions" OR "multiple chronic diseases" OR "multiple conditions" OR "multiple diseases" OR "multiple disorders" OR polymorbid* OR "poly-morbid" OR "poly-morbidity" OR "poly-morbidities" OR "poly morbid" OR "poly morbidity" OR "poly morbidities" OR polypath* OR pluripath* OR multipath* OR "multi pathology" OR "multi pathologies" OR "multi-pathology" OR "multi-pathologies" OR "multiple pathologies" OR "disease cluster" OR "disease clusters" OR "disease pattern" OR "disease patterns" OR "concurrent chronic diseases" OR "multiple chronic disorders" OR Comorbidity [DE]

S4 Frailty OR frail

S5

"covid-19" OR "2019-ncov" OR "sars cov 2" OR "cov-19" OR cov19 OR "cov 19" OR 2019nCoV OR betacoronavirus* OR "corona virus" OR coronavirus* OR coronovirus* OR CoV OR CoV2 OR COVID OR covid19 OR "covid 2019" OR "Severe Acute Respiratory Syndrome Coronavirus 2" OR "2019 ncov" OR (wuhan AND Coronavirus) OR "HCoV-19" OR nCoV OR SARS2 OR SARSCoV OR (sars AND virus) OR "sars virus" OR (sars AND CoV) OR "sars cov" OR "severe acute respiratory syndrome cov" OR "SARS CoV-2" OR "SARS-

For peer review only - http://bmjopen.bmj.com/site/about/guidelines.xhtml

PsycINFO strategy

CoV-2" OR SARSCoV2 OR "CoV-2" OR "covid 19" OR covid2019 OR "covid-2019" OR "novel CoV" OR "corona pandemic" OR "wuhan virus" OR "CoV 2" OR ((wuhan OR hubei OR huanan) AND ("severe acute respiratory" OR pneumonia*) AND outbreak*) OR COVID-19 [DE]

S6

(S2 OR S3 OR S4) AND S5 AND S1

For peer review only

Embase strategy

((comorbid* OR 'comorbidity'/exp OR comorbidity OR 'multiple chronic conditions'/exp OR 'multiple chronic conditions' OR multimorbid* OR 'multi morbid' OR 'multi morbidity' OR 'multi morbidities' OR 'co morbid' OR 'co morbidity' OR 'co morbidities' OR 'disease pattern' OR 'disease patterns' OR 'disease cluster' OR 'disease clusters' OR 'multiple conditions' OR 'multiple diseases' OR 'multiple disorders' OR polymorbid* OR 'poly morbid' OR 'poly morbidity' OR 'poly morbidities' OR polypath* OR 'multiple pathology' OR 'multiple pathologies') OR ('frail elderly'/exp OR 'frail elderly' OR 'frailty'/exp OR 'frailty' OR frail*) OR (('socioeconomics'/exp OR 'socioeconomics' OR 'social status'/exp OR 'social status' OR 'social stratification'/exp OR 'social stratification' OR 'social class'/exp OR 'social class') OR ('urban area'/exp OR 'urban area' OR 'rural area'/exp OR 'rural area') OR 'population group'/exp OR ('minority group'/exp OR 'minority group' OR 'homelessness'/exp OR 'homelessness' OR 'migrant'/exp OR 'migrant' OR 'race'/exp OR 'race' OR ethnic* OR 'minority groups') OR ('economic status'/exp OR 'economic status' OR 'income'/exp OR 'income' OR 'income group'/exp OR 'income group') OR ('social discrimination'/exp OR 'social discrimination' OR 'socioeconomic inequality'/exp OR 'socioeconomic inequality' OR 'health disparity'/exp OR 'health disparity' OR 'poverty'/exp OR 'poverty' OR 'income inequality'/exp OR 'income inequality') OR ('educational status'/exp OR 'educational status' OR 'literacy'/exp OR 'literacy' OR 'education* level') OR ('family size'/exp OR 'family size' OR 'household characteristics' OR 'household size' OR 'household'/exp))) AND ('longitudinal study'/exp OR 'longitudinal study' OR 'cross-sectional study'/exp OR 'cross-sectional study' OR longitudinal OR 'cross sectional' OR 'case control study'/exp OR 'case control study' OR 'case control' OR cohort OR 'ecological study' OR 'ecological design' OR 'observational study'/exp OR 'observational study' OR 'retrospective study'/exp OR 'retrospective study' OR 'prospective study'/exp OR 'prospective study' OR 'prospective observational study' OR 'prospective observational studies' OR 'retrospective observational study' OR 'retrospective observational studies' OR 'ecological designs' OR 'prospective studies' OR 'retrospective studies' OR 'observational studies' OR 'ecological studies') AND ('coronavirus disease 2019'/exp OR 'coronavirus disease 2019' OR 'covid 2019' OR 'covid 19' OR covid2019 OR covid19 OR sarscov2 OR 'sars cov 2' OR 'sars cov2' OR 'severe acute respiratory syndrome'/exp OR 'severe acute respiratory syndrome' OR 'wuhan virus' OR 'cov 2' OR cov2 OR coronavirus OR 'corona virus' OR 'novel cov' OR 2019ncov OR '2019 ncov' OR 'corona pandemic' OR betacoronavirus OR cov19 OR 'cov 19' OR 'hcov-19' OR ncov OR sars2) AND [english]/lim AND [2020-2021]/py

For peer review only

PRISMA-S Checklist

Section/topic	#	Checklist item	Location(s) Reported
**INFORMATION SOURCES AND METHODS**			
Database name	1	Name each individual database searched, stating the platform for each.	Page 7
Multi-database searching	2	If databases were searched simultaneously on a single platform, state the name of the platform, listing all of the databases searched.	NA
Study registries	3	List any study registries searched.	NA
Online resources and browsing	4	Describe any online or print source purposefully searched or browsed (e.g., tables of contents, print conference proceedings, web sites), and how this was done.	NA
Citation searching	5	Indicate whether cited references or citing references were examined, and describe any methods used for locating cited/citing references (e.g., browsing reference lists, using a citation index, setting up email alerts for references citing included studies).	Page 8
Contacts	6	Indicate whether additional studies or data were sought by contacting authors, experts, manufacturers, or others.	Page 8
Other methods	7	Describe any additional information sources or search methods used.	NA
**SEARCH STRATEGIES**			
Full search strategies	8	Include the search strategies for each database and information source, copied and pasted exactly as run.	Page 7 and Annex 1
Limits and restrictions	9	Specify that no limits were used, or describe any limits or restrictions applied to a search (e.g., date or time period, language, study design) and provide justification for their use.	Page 8
Search filters	10	Indicate whether published search filters were used (as originally designed or modified), and if so, cite the filter(s) used.	NA
Prior work	11	Indicate when search strategies from other literature reviews were adapted or reused for a substantive part or all of the search, citing the previous review(s).	Page 7
Updates	12	Report the methods used to update the search(es) (e.g., rerunning searches, email alerts).	Page 8

For peer review only

Dates of searches	13	For each search strategy, provide the date when the last search occurred.	Page 8
**PEER REVIEW**			
Peer review	14	Describe any search peer review process.	Pages 8, 9, 10
**MANAGING RECORDS**			
Total Records	15	Document the total number of records identified from each database and other information sources.	Page 10
Deduplication	16	Describe the processes and any software used to deduplicate records from multiple database searches and other information sources.	Page 8

PRISMA-S: An Extension to the PRISMA Statement for Reporting Literature Searches in Systematic Reviews
Rethlefsen ML, Kirtley S, Waffenschmidt S, Ayala AP, Moher D, Page MJ, Koffel JB, PRISMA-S Group.
Last updated February 27, 2020.

For peer review only

BMJ Open

Etiologic and prognostic roles of frailty, multimorbidity and socioeconomic characteristics in the development of SARS-CoV-2 health outcomes: protocol for systematic reviews of population-based studies

Journal:	*BMJ Open*
Manuscript ID	bmjopen-2022-063573.R1
Article Type:	Protocol
Date Submitted by the Author:	31-Aug-2022
Complete List of Authors:	Makovski, Tatjana; Sante publique France,<br>Ghattas, Jinane; UCLouvain<br>Monnier Besnard, Stephanie; Sante publique France<br>Ambrozova, Monika; Institute of Health Information and Statistics of the Czech Republic<br>Vasinova, Barbora; Institute of Health Information and Statistics of the Czech Republic<br>Feteira-Santos , Rodrigo ; Universidade de Lisboa, Instituto de Saúde Ambiental, Faculdade de Medicina<br>Bezzegh, Peter; Government of Hungary<br>Ponce Bollmann, Felipe; Universidad Nacional de Educación a Distancia<br>Cottam, James; Sciensano<br>Haneef, Romana; Sante publique France<br>Devleesschauwer, Brecht; Sciensano, Epidemiology and public health<br>Speybroeck, Niko; Université catholique de Louvain, Institut de recherche santé et société (IRSS)<br>Nogueira, Paulo; Universidade de Lisboa Faculdade de Medicina<br>Forjaz, Maria João; National School of Public Health and Health Service Research Network for Chronic Diseases (Red de Investigación en Servicios de Salud en Enfermedades Crónicas/REDISSEC), Carlos III Institute of Health.<br>Coste, Joel; Sante publique France<br>Carcaillon-Bentata, Laure; Sante publique France
**Primary Subject Heading**:	Epidemiology
Secondary Subject Heading:	Public health
Keywords:	COVID-19, EPIDEMIOLOGY, PUBLIC HEALTH

SCHOLARONE™
Manuscripts

Etiologic and prognostic roles of frailty, multimorbidity and socioeconomic characteristics in the development of SARS-CoV-2 health outcomes: protocol for systematic reviews of population-based studies

Authors

Tatjana T. Makovski[1], Jinane Ghattas[2], Stéphanie Monnier-Besnard[1], Monika Ambrožová[3], Barbora Vašinová[3], Rodrigo Feteira-Santos[4,11], Peter Bezzegh[5], Felipe Ponce Bollmann[6], James Cottam[7,10], Romana Haneef[1], Brecht Devleesschauwer[7,9], Niko Speybroeck[2], Paulo Jorge Nogueira[4,11], Maria João Forjaz[8], Joël Coste[1], Laure Carcaillon-Bentata[1]

Authors' affiliations

[1] Public health France, France

[2] Institute of Health and Society, Université catholique de Louvain, Belgium

[3] Institute of Health Information and Statistics of the Czech Republic, Czech Republic

[4] Área Disciplinar Autónoma de Bioestatística, Faculdade de Medicina, Universidade de Lisboa, Lisboa, Portugal

[5] Directorate for Project Management, National Directorate General for Hospitals , Hungary

[6] Universidad Nacional de Educación a Distancia, Spain

[7] Department of Epidemiology and Public Health, Sciensano, Brussels, Belgium

[8] National Center of Epidemiology, Instituto de Salud Carlos III, REDISSEC and RICAPPS, Spain

[9] Department of Translational Physiology, Infectiology and Public Health, Ghent University, Merelbeke, Belgium

[10] Institute of Tropical Medicine, Antwerp, Belgium

[11] Instituto de Saúde Ambiental, Faculdade de Medicina, Universidade de Lisboa, Lisboa, Portugal

Corresponding author

Tatjana T. Makovski

Agence nationale de santé publique
12 rue du Val d'Osne
94415 Saint-Maurice Cedex France
www.santepubliquefrance.fr

tatjanamakovski@hotmail.com

For peer review only

Abstract

Introduction: There is growing evidence that the impact of COVID-19 crisis may be stronger for individuals with multimorbidity, frailty and lower socioeconomic status. Existing reviews focus on few, mainly short-term effects of COVID-19 illness and patients with single chronic disease. Information is also largely missing for population representative samples.

Applying population-based approach, the systematic reviews will have two objectives, to: 1) evaluate the etiologic roles of frailty, multimorbidity and socioeconomic status on SARS-CoV-2 infection probability, hospitalization, intensive care unit (ICU) admission, mechanical ventilation and COVID-19 related mortality among general population; 2) investigate the prognostic roles of frailty, multimorbidity and socioeconomic characteristics on the risk of hospitalization, ICU admission, mechanical ventilation, COVID-19 mortality, functioning, quality of life, disability, mental health and work absence.

Methods and analysis: For this ongoing work, four databases were searched: PubMed, Embase, WHO COVID-19 Global literature on coronavirus disease and PsycINFO, for the period between January 2020 and April 7 2021. Peer-reviewed published literature in English will be considered and all types of population-based studies. Studies using standard tools to assess multimorbidity such as disease count, comorbidity indices or disease combinations will be retained, as well as studies with standard scales and scores for frailty or measurement of a socioeconomic gradient. Initial search included 10,139 articles, 411 for full-text reading. Results will be summarized by risk factor, objective and outcome. The feasibility of meta-analysis will be determined by the findings and will aim to better understand uncertainties of the results. Quality of studies will be assessed using standardized scales.

Ethics and dissemination: The study will be based on published evidence and it is exempt from the ethical approval. This work is part of the Population Health Information Research Infrastructure (PHIRI) project. Dissemination of the results will imply conference presentation, submission for scientific publication and PHIRI project report.

Prospero registration: CRD42021249444.

For peer review only

Strengths and limitations of this study

- This is a study protocol of systematic reviews on the etiologic and prognostic roles of multimorbidity, frailty and socioeconomic characteristics on development of short and long-term SARS-CoV-2 health outcomes.
- Only quantitative studies conducted on population representative samples will be eligible.
- Only peer-reviewed literature published in English will be considered.
- The literature screening process, data extraction and study quality assessment will be performed by two reviewers independently.
- The findings will be summarized by risk factor, etiologic or prognostic role and study outcome.

For peer review only

For peer review only - http://bmjopen.bmj.com/site/about/guidelines.xhtml

Introduction

The early months of 2020 were marked with rapid growth in infections caused by the novel severe acute respiratory syndrome coronavirus 2 or SARS-CoV-2, leading to the state of a global pandemic declared by the World Health Organization (WHO) on March 11, 2020 (1). SARS-CoV-2 causes coronavirus disease 2019 (COVID-19) (2), which has led to severe health and health care management consequences around the world (3).

It has been demonstrated so far that age, male sex and comorbidity are the main risk factors for COVID-19 hospitalisations, intensive care unit (ICU) admissions and mortality (4-6). Specifically, infection fatality rates due to COVID-19 have been found to exponentially increase with age : 0.4% at age 55, 1.4% at age 65, 4.6% at age 75 and 15% at age 85 (7). In addition, having at least one comorbidity more than doubled the risk of hospitalisation while certain comorbidities, including severe kidney disease, diabetes, severe immunodeficiency or heart failure, among few others, increased this risk in particular, e.g., odds ratio (OR)/hazard ratio (HR) ≥ 3 for hospitalisation or death (8). Much remains to be clarified due to the lifespan of the new disease of only two years and the appearance of new virus variants (9) against which the same protection level conferred by existing vaccines is not guaranteed (10). In particular, regarding which and why certain population groups still display more severe symptoms and outcomes compared to others.

Frailty, as a marker of increased vulnerability due to accumulated deficits in multiple body systems (11) and multimorbidity, commonly defined as having two or more chronic conditions (12) are well recognized risk factors for adverse health outcomes in the elderly population, including hospitalisation, dependency and mortality (13-15). In the context of the COVID-19 pandemic, it is plausible that both frailty and multimorbidity increase the risk of SARS-CoV-2 infection and the development of severe outcomes by altering older people's biological background and immunity. Some studies have found that frailty and multimorbidity predicted COVID-19 patients' prognosis for hospitalisation, mortality and level of care at discharge (16-26). To date, however, most studies addressing these relationships are likely to be biased due to the inclusion of only severe COVID-19 cases admitted to the hospital. Whether the relationships of frailty and multimorbidity with COVID-19 infection and outcomes hold for non-severe forms of COVID-19, it is of major interest to improve risk stratification and public health guidance in the general population (27).

Genetic and biomedical factors are not the only determinants that may influence COVID-19 outcomes. During previous pandemics, lower socioeconomic status was associated with a higher disease burden. It is therefore important to consider social vulnerability (28). Several studies suggest that COVID-19, like other infectious diseases, disproportionally affects socially disadvantaged population groups and places them at a higher risk of disease severity. People of low socioeconomic status, ethnic minority groups (29) and crowded households (30) had higher risks of acquiring COVID-19 and higher hospitalisation rates. Overall, a large number of studies with very different methodological approaches (settings, type of adjustment) have been conducted to assess the role of socioeconomic factors in SARS-CoV-2 infection and outcomes. Therefore, it is important to synthesize the current knowledge from well-designed population-based studies in order to clarify and quantify the weight of each socioeconomic factor.

For peer review only

Finally, most of the investigated COVID-19 related health impacts appear so far to be of short-term, e.g. hospitalisations, ICU admissions or mortality. Evidence on long-term effects such as potential mental health difficulties, impact on quality of life or functioning, among others, in COVID-19 patients is limited (31, 32), but it will certainly grow as the pandemic prolongs.

The above assumptions justify the relevance, need and importance of summarizing current knowledge using a systematic review approach regarding the etiologic and prognostic roles of frailty, multimorbidity and socioeconomic characteristics in developing COVID-19 disease and related short and long-term health consequences, among general population. The Prospero registry for systematic reviews (33) was cross-checked to ensure the novelty of the research questions.

Objectives

This paper aims to describe the methodology, which will be used for conducting systematic reviews intended at synthesising knowledge on the roles of frailty, multimorbidity and socioeconomic characteristics in the acquisition and evolution of COVID-19 related health outcomes. Two main study objectives have been determined.

The first objective is to explore the effects of frailty, multimorbidity and socioeconomic characteristics on SARS-CoV-2 infection risk in the general population. The goal is to consider the general population at risk of developing SARS-CoV-2 infection or related outcomes and to estimate a potential increase in risk associated with a given risk factor (frailty or multimorbidity or socioeconomic characteristics). The outcomes of interest are infection by SARS-CoV-2, hospitalisation for COVID-19, ICU admission, mechanical ventilation and mortality due to COVID-19.

The second objective is to explore the prognostic effects of frailty, multimorbidity and socioeconomic characteristics in SARS-CoV-2 infected subjects from the general population. The goal is to consider SARS-CoV-2 infected individuals from the general population and estimate a potential increase in risk of developing severe short- and long-term outcomes associated with a given risk factor (frailty, multimorbidity or socioeconomic characteristics). The outcomes of interest are death due to COVID-19, hospitalization, ICU admission, mechanical ventilation, functioning, quality of life, disability, mental health difficulties and work absence/sick leave.

For peer review only

Methods and analysis

Protocol and registration

This protocol is written along the requirements of Preferred Reporting Items for Systematic Review and Meta-Analysis Protocols (PRISMA-P) 2015 checklist (34). The study is registered in the Prospero registry for systematic review protocols under the registration number: CRD42021249444.

Should there be any substantial amendments to this protocol in the course of the study, they will be fully documented and included in dissemination, such as reports and scientific publications.

Eligibility criteria

We applied the Population, Exposure, Comparator and Outcomes (PECO) framework (35) to clearly specify research objectives. This framework has been increasingly recognized in population health, environmental and

occupational health fields due to the importance of identifying the exposure and clarifying its association with the outcome using comparator groups; (35, 36). The study objectives are hence further detailed as follows:

Objective 1

P Population = general or well-defined population SARS-CoV-2 negative

E Exposure = (either) frailty, multimorbidity, lower socioeconomic status

C Comparator = general population with no frailty or no multimorbidity or with better socioeconomic status

O Outcome(s) = infection by SARS-COV-2, hospitalization for COVID-19, ICU admission, mechanical ventilation, mortality due to COVID-19

Objective 2

P Population = general or well-defined population SARS-CoV-2 infected (COVID-19 diagnosed, with e.g. polymerase chain reaction (PCR) test, medical imaging or similar)

E Exposure = (either) frailty, multimorbidity, lower socioeconomic status

C Comparator = SARS-CoV-2 infected subjects from the population with no frailty or no multimorbidity or with better socioeconomic status

O Outcome(s) = death due to COVID-19, hospitalization for COVID-19, ICU admission, mechanical ventilation, functioning, quality of life, disability, mental health difficulties and work absence/sick leave.

A widely agreed definition of multimorbidity is lacking in the scientific literature (37, 38), however the most frequently cited definition is a coexistence of two or more conditions within an individual (12, 38) which will likewise apply in this review. Multimorbidity can also be operationalised differently, in a sense that it is commonly explored as a disease count (≥ 2, ≥ 3 diseases or similar), through comorbidity/multimorbidity indices or disease combinations. To summarize all relevant evidence, studies with any of the valid mentioned measurement tools that observe the association between minimum of two conditions and COVID-19 related outcomes will be retained. In the final summary table, this review will specify the multimorbidity measurement tool, and the number and list of conditions for each included study.

Similarly, number of definitions for frailty can be found in the literature where each holds its advantages and limitations. Two main approaches coexist and are widely used to define frailty: the phenotypic approach described by Fried et al. in 2001 (13) and the deficit accumulation approach proposed by Rockwood et al. in 2004 (14). We choose to include all studies using a definition of frailty based on one of these two approaches, provided that its predictive value had been previously validated. Studies using the term "frailty" without reference to either of these two standards will not be included.

Likewise, literature looking at the role of the socioeconomic gradient in COVID-19 related outcomes will be retained. Socioeconomic status assessment will include standard indicators, such as income, education, occupation, employment, housing, urban/rural setting, household size, race and marital status measured at the individual or the community level (ecological studies). All age groups will be taken into account considering that socioeconomic differences may be observed across all ages and that multimorbidity is increasingly present among younger individuals as well as among older adults (39).

For peer review only - http://bmjopen.bmj.com/site/about/guidelines.xhtml

Eligible studies and settings

To identify population based studies, the following definitions will apply: "a population-based study is defined as a study of properties of a well-defined population, such as individuals residing in a defined geographic region in a given time period"(40) and "population-based studies aim to answer research questions for defined populations; answers should be generalizable to the whole population addressed in the study hypothesis, not only to the individuals included in the study" (41). Therefore, the following examples of population-based studies may be eligible for our reviews: community-dwelling, hospital-based, nursing homes, homes for elderly, or similar, as long as the population in the study is representative of the country or region from which the sample was drawn. Only quantitative studies will be considered.

Study designs

All types of etiologic and prognostic observational studies with comparator groups will be eligible, such as cohorts, cross-sectional studies, case-control studies or ecological studies (for socioeconomic determinants only). Interventions and clinical trials will be excluded, as well as qualitative and case studies. Only published peer-reviewed original studies written in English will be considered.

Information sources and search strategy

The following databases were be explored: PubMed, Embase, WHO COVID-19 Global literature on coronavirus disease (42) and PsycINFO. The search strategy considers all possible variations of the terms: multimorbidity, frailty, terms defining socioeconomic characteristics, COVID-19 and study design types. The search strategy was adjusted to the technical specificities of each database. The example of the search strategy in PubMed is presented in Table 1. The search strategy for remaining three databases is presented in Annex 1.

Table 1 PubMed search strategy

#1	Covid-19 (Adjusted from Lazarus et al.)(43)	((((((((((((((((((("Betacoronavirus"[MeSH Terms] OR "Coronavirus Infections"[MeSH Terms]) OR "COVID-19"[Supplementary Concept]) OR "Coronavirus"[MeSH Terms]) OR "Severe Acute Respiratory Syndrome Coronavirus 2"[Supplementary Concept]) OR "2019nCoV"[All Fields]) OR "betacoronavirus*"[All Fields]) OR "corona virus*"[All Fields]) OR "coronavirus*"[All Fields]) OR "coronovirus*"[All Fields]) OR "CoV"[All Fields]) OR "CoV2"[All Fields]) OR "COVID"[All Fields]) OR (("COVID-19"[Supplementary Concept] OR "COVID-19"[All Fields]) OR "covid19"[All Fields])) OR (((((("COVID-19"[All Fields] OR "covid 2019"[All Fields]) OR "Severe Acute Respiratory Syndrome Coronavirus 2"[Supplementary Concept]) OR "Severe Acute Respiratory Syndrome Coronavirus 2"[All Fields]) OR "2019 ncov"[All Fields]) OR "SARS CoV 2"[All Fields]) OR "2019nCoV"[All Fields]) OR (("wuhan"[All Fields] AND ("Coronavirus"[MeSH Terms] OR "Coronavirus"[All Fields])) AND (2019/12/1:2019/12/31[Date - Publication] OR 2020/1/1:2020/12/31[Date - Publication])))) OR "HCoV-19"[All Fields]) OR "nCoV"[All Fields]) OR "SARS CoV 2"[All Fields]) OR "SARS2"[All Fields]) OR "SARSCoV"[All Fields]) OR (((("sars virus"[MeSH Terms] OR ("sars"[All Fields] AND "virus"[All Fields])) OR "sars virus"[All Fields]) OR ("sars"[All Fields] AND "CoV"[All Fields])) OR "sars cov"[All Fields])) OR (("Severe Acute Respiratory Syndrome Coronavirus 2"[Supplementary Concept] OR "Severe Acute Respiratory Syndrome Coronavirus 2"[All Fields]) OR "SARS CoV 2"[All Fields])) OR "severe acute respiratory syndrome cov*"[All Fields]) AND

For peer review only

		(2019/11/17:3000/12/31[Date - Entry] OR 2019/11/17:3000/12/31[Date - Publication]) OR "COVID-19"[MeSH Terms] OR "SARS-Cov-2"[MeSH Terms] OR "SARS CoV-2" OR "SARS-CoV-2" OR SARSCoV2 OR "CoV-2" OR "covid 19" OR covid2019 OR "covid-2019" OR "novel CoV" OR "corona pandemic*" OR "wuhan virus*" OR "CoV 2" OR ((wuhan OR hubei OR huanan) AND ("severe acute respiratory" OR pneumonia*) AND outbreak*)
#2	Frailty	frailty OR frail OR frailty[MeSH Terms]
#3	Multimorbidity (Adjusted from Makovski et al.)(44)	multimorbidity OR "multi-morbidity" OR "multi morbidity" OR multimorbidities OR "multi-morbidities" OR "multi morbidities" OR multimorbid OR "multi-morbid" OR "multi morbid" OR comorbidity OR "co-morbidity" OR "co morbidity" OR comorbidities OR "co-morbidities" OR "co morbidities" OR comorbid OR "co morbid" OR "multiple chronic conditions" OR "multiple chronic diseases" OR "multiple conditions" OR "multiple diseases" OR "multiple disorders" OR polymorbid* OR "poly-morbid*" OR "poly morbid*" OR polypath* OR pluripath* OR multipath* OR "multi path*" OR "multi-path*" OR "multiple pathologies" OR "disease cluster" OR "disease clusters" OR "disease pattern" OR "disease patterns" OR "concurrent chronic diseases" OR "multiple chronic disorders" OR multimorbidity[MeSH Terms] OR comorbidity[MeSH Terms]
#4	Socioeconomic characteristics	"socio economic" OR "socio-economic" OR socioeconomics OR "socio-economics" OR "socio economics" OR socioeconomic OR "social difference" OR "social differences" OR "social inequality" OR "social inequalities" OR "socioeconomic inequality" OR "socioeconomic inequalities" OR "social disparity" OR "social disparities" OR education OR literacy OR "socioprofessional" OR "socio-professional" OR "socio professional" OR "social conditions" OR "social class" OR "social classes" OR "social class"[MeSH Terms] OR "socioeconomic factors"[MeSH Terms] OR health status disparities[MeSH Terms] OR income OR poverty OR deprivation OR rural OR urban OR "housing deprivation" OR homeless OR houseless OR homelessness OR ethnic* OR race OR emigrant OR immigrant OR migrant OR "minority group" OR "minority groups" OR disadvantaged OR "marital status" OR ((characteristics OR factors OR status) AND (economic OR social OR educational)) OR ((composition OR characteristics OR size) AND (family OR household))
#5	Study design	"cross-sectional" OR "cross sectional" OR "case-control" OR "case control" OR cohort OR longitudinal OR "ecological study" OR "ecological studies" OR "ecological design" OR "ecological designs" OR observational OR "observational study" OR "observational studies" OR "observational design" OR "observational designs" OR "prospective study" OR "prospective studies" OR "prospective design" OR "prospective designs" OR "retrospective study" OR "retrospective studies" OR "retrospective design" OR "retrospective designs" OR "prospective observational study" OR "prospective observational studies" OR "retrospective observational study" OR "retrospective observational studies" OR case-control studies[MeSH Terms] OR cohort studies[MeSH Terms] OR cross-sectional studies[MeSH Terms]
#6		#2 OR #3 OR #4
#7		#1 AND #5 AND #6

The search was restricted using date (January 2020 to April 7, 2021) and language (English) filters. Reference lists of retained studies will be explored for potentially omitted relevant records. For studies where pertinent data are not presented but seem to be available or if measurements of interest (SARS-CoV-2 diagnosis, outcomes and

For peer review only

For peer review only - http://bmjopen.bmj.com/site/about/guidelines.xhtml

risk factors) are not clearly defined, study authors will be contacted for details. If deemed relevant, update of the literature search will be performed.

Study records

Data management

The literature will be managed using Rayyan (45), EndNote and Excel. Records retrieved from the four databases will be exported in EndNote for deduplication. Title and abstract screening will be performed in Rayyan, a web and mobile app for systematic reviews. Full-text articles will be read in EndNote and data will be extracted in customised Excel tables. Two researchers will perform the screening process and data extraction. Considering the actuality of the topic, the quantity of the literature envisaged and the need for timely information, several researchers will be involved in the screening process. For example, two researchers will assume the role of the first reviewer while several colleagues will act as the second reviewer. Weekly meetings will ensure a clear understanding of the study process. In addition, prior to the start of the study selection, a pilot test is foreseen; for instance, homogeneity in the understanding of the exclusion and inclusion criteria among reviewers will be compared and explained on a minimum of 50 studies.

For peer review only

Selection process

Applying the exclusion criteria displayed in List 1, two reviewers will independently screen all records by title and abstract (records screening phase) to select studies for full-text reading (reports screening phase) (46).

List 1: Reasons for exclusion from the systematic review (title/abstract or records screening phase)

1 = Language other than English

2 = Not an original research (e.g. editorial, protocol, etc.) or no original results

3 = Unrelated topic

4 = Not a population-based study

5 = Subpopulation (medical staff, students, pregnant women, etc.)

6 = Duplicate

The criteria above will be used in a hierarchical order to reduce the discrepancy in selected exclusion criteria between the reviewers, implying that should there be more than one criterion applicable to a study, the one higher on the list will be assigned.

Exclusion criteria in List 2 will apply for selecting studies during a full-text (reports) reading phase.

List 2: Reasons for exclusion from the systematic review (full-text reading or reports screening phase)

1 = Not a population-based study

2 = SARS-CoV-2 infection diagnosis not clear*

For peer review only - http://bmjopen.bmj.com/site/about/guidelines.xhtml

3 = Study does not consider people with frailty or multimorbidity OR does not contain information on socioeconomic characteristics

4 = Outcome not within the scope of our objectives

5 = Outcome measurement tool not clear*

6 = Risk factor measurement tool not clear*

7 = Subpopulation (e.g. pregnant women, health care workers, students etc.)

8 = Not an original research (e.g. editorial, protocol, review, conference abstract, grey literature, etc.), no original results or not peer-reviewed

9 = The same or largely the same population already considered in another study for the same outcome

10 = Clinical trial or intervention study

11 = Qualitative study

12 = Descriptive study, absence of a comparator group and/or no measure of the association of interest

13 = Other (explain)

*even after contacting authors

The records will be rated as eligible/not eligible/questionable (47) at both screening stages. Any disagreement between the reviewers will be resolved by consensus in referral to this protocol; a third reviewer will be consulted if necessary. Disagreement between the reviewers will be reported in percentages (48).

During the screening process, all articles will be sorted by type of exposure factors, e.g. frailty and multimorbidity on one side (as biomedical determinants) and socioeconomic characteristics on the other, and by objective (objective 1 or 2). Articles on biomedical determinants will later be divided to studies on frailty or multimorbidity. Retained studies will be kept for further analyses.

Data items

Six extraction tables for each exposure and objective will be prepared. They will include: study title, authors, year of publication, study design, study setting and duration, population, sample size, COVID-19 diagnostic criteria, risk factors and study outcomes and means by which they were assessed, measurement of the association between variables of interest, and study adjustment factors.

Two reviewers will extract data independently and compare for agreement (48).

Risk of bias in individual studies

Two reviewers will separately assess a risk of bias for each study retained for final reporting. Any disagreement will be resolved by consensus; otherwise, a third reviewer will be consulted. The Newcastle-Ottawa scales will be used to assess the quality of case-control and cohort studies (49). Each of these scales contains eight items grouped in three categories: selection, comparability and outcome or exposure (for cohort and case-control studies, respectively). Selection items refer to e.g. representativeness of the sample, ascertainment of the exposure, definitions of case and control group, etc. Comparability items inquire on comparability between e.g. cases and controls or between cohorts based on the study design or analysis. Outcomes category items account for the assessment of the outcome or adequacy of the follow up, while exposure items account for e.g.

For peer review only

ascertainment of the exposure or response rate. Each scale item can carry a maximum of one point, except for the comparability group, where up to two points for an item can be assigned, totalling a maximum of nine points for study quality. Higher score indicates higher study quality.

An adjusted version of the Newcastle-Ottawa scale will be used to assess quality for cross-sectional studies (50, 51). Here maximum of 10 points can be assigned.

Abovementioned scales will also apply to assess study quality of ecological studies (for socioeconomic risk factor only), depending of the ecological study design.

Data synthesis

The literature review is ongoing. The search included 10,139 articles in title and abstract screening phase, of which 411 were retained for full-text reading. The screening process results will be presented with PRISMA diagram (52). Given the interest in several risk factors, the PRISMA diagram may be adjusted, as suggested in Fig. 1.

Fig. 1 PRISMA 2020 flow diagram for new systematic reviews, which included searches of databases and registers only (adjusted)

**BM = Biomedical, SE= Socioeconomic*

The final set of retained studies will be described using standardised tables. They will be summarised within each objective, by type of risk factor and study outcome and presented in tabulated form. Study characteristics will be described as well as their main findings.

When possible, meta-analysis models will be performed by each objective, for each risk factor and for a specific outcome with the aim to better understand the uncertainties (random error and bias) of the obtained results. The feasibility of performing meta-analyses will depend on additional criteria, such as number of studies applying the same measurement tools for risk factors and evaluating the same outcomes, as well as reporting odds ratio, hazard ratio, risk ratio or slope. For the meta-analysis, a minimum of four studies will be required. In studies assessing the role of socioeconomic inequalities on health outcomes, unadjusted studies stratified by age group will be sought to avoid overadjustment (53). For studies assessing the roles of biomedical factors, adjusted analyses will be preferred. Adjustment factors will be thoroughly reported. Random effects meta-analyses will be performed with R software using the metafor package (54). Findings will be presented with forest plot(s). Publication bias will be visually assessed using funnel plots and formally tested using Begg and Mazumdar's rank correlation test (55) and Egger's regression test (56). When significant publication bias will be present, Duval and Tweedie's trim-and-fill method will be used to explore the impact on model estimates (57). Heterogeneity will be explored by computing I^2 statistic (58). When feasible, sensitivity analysis or meta-regression using study quality as a covariate will be conducted. Other covariates such as proper operationalisation of multimorbidity or frailty (e.g. number and list of diseases clearly provided or proper definition used), mean age or gender proportion, may also be considered.

For peer review only - http://bmjopen.bmj.com/site/about/guidelines.xhtml

Patient and public involvement

Patients and/or the public were not involved in the design of the protocol and will not be involved in conducting the study, neither in reporting or dissemination plans of the research.

Discussion

This protocol aims to present the methodology that will be applied to systematically assess the evidence on the etiologic and prognostic roles of multimorbidity, frailty and socioeconomic risk factors in the development of SARS-CoV-2 infection and COVID-19 related severe short- and long-term health outcomes, among the general population. These reviews should inform on the quantity and quality of the relevant literature and summarise the existing evidence. In addition, the reviews will report which were the preferred assessment methods for the risk factors and most frequent study outcomes considered, as well as which were the statistical approaches and adjustment factors across studies.

To date, no systematic review on the link between multimorbidity and SARS-CoV-2 infection and outcomes has been identified. Even tough not specifically focusing on patients with multimorbidity, Kuswardhani et al. (59) have summarised evidence on the association between the Charlson's score and poor short-term outcomes in COVID-19 patients. This score is still frequently used to assess health status in patients with multiple conditions, in the absence of a more specific and widely accepted multimorbidity tool. We plan to elaborate on other multimorbidity measurements and broader COVID-19 related consequences, and investigate the possible etiologic role of multimorbidity in the illness acquisition and development. In the context of ageing societies where an increasing number of people live with multiple conditions, scarcity of evidence and public health advice for this population group in the COVID-19 crisis is not acceptable. It is essential to inform how to best evaluate the risks for people with multiple number and combinations of diseases to provide clinicians and policy makers with adequate advice for the most optimal and evidence-based decisions (27).

On the other hand, several systematic reviews have been conducted among frail population (22, 25). However, these focus on hospital settings, usually single centre or few multicentre settings and short-term outcomes; prognostic roles of frailty are also mainly explored. None of the identified reviews investigated the role of frailty in the development of COVID-19 related outcomes in population representative samples. Our systematic review intends to build on the existing evidence by examining the occurrence of a wide number of COVID-19 health outcomes accounting for etiologic as well as prognostic roles of frailty, among the general population.

Similarly, reviews show existing disparities in COVID-19 outcomes in lower socioeconomic groups and ethnic minorities. Specific contexts such as low level of education, poor housing conditions and crowded households could play a role in worsening COVID-19 outcomes (60-62). As above, no systematic review to date has investigated COVID-19 outcomes and severity related to socioeconomic gradient in well-defined population based studies. We, therefore aim to fill this gap. Whereas the three types of factors (multimorbidity, frailty and socioeconomic factors) will be considered and analyzed separately, the similar use of a population-based approach justify the definition of a common methodology and the sharing of the first steps of the reviews performed.

For peer review only - http://bmjopen.bmj.com/site/about/guidelines.xhtml

A first limitation of these systematic reviews is the focus on quantitative studies, using comparator groups and providing measures of associations between considered exposures and outcomes. Case and qualitative studies, which may be informative of more complex connections between multimorbidity, frailty, socioeconomic status, and COVID-19, but whose evidence synthesis require the use of methodologies different from systematic reviews of quantitative studies (63-65), will be left aside for the sake of feasibility. A second limitation of this review more specifically concerns socioeconomic status indicators which are often relational and contextual (to a country, a region, a city) and that heterogeneity may result from their use, precluding meta-analyses in several cases. A third potential limitation of the reviews is that only publications written in English will be considered. With this, we possibly risk to overlook national population representative studies, which may have been in the necessity of the crisis as well as for local purposes, released in local languages exclusively. On the other hand, a publication in peer-reviewed international journals in English may also be regarded as an additional study quality check. A strength and a limitation could be the number of scientists who are planned for this study. Involving several team members may accelerate the review process, which is desired when information is crucial. However, one needs to consider the heterogeneity among reviewers in understanding the study method and the review criteria. We will overcome this by conducting a pilot test and holding regular team and individual meetings with involved parties. Moreover, the two first reviewers, experts in respectful areas, will remain the same throughout the screening and data extraction process. Accurately following validated guidelines in preparing this protocol, conducting and reporting the study will help to limit any possible biases to a minimum. It is worth to mention a high quantity and speed of which the literature is produced during the COVID-19 crisis, as well as a high pace at which data become obsolete. Our reviews intend to provide some of the first estimates of the association between the chosen risk factors and various COVID-19 related outcomes at the population level, by compiling evidence published in a limited timeframe. We will critically and prudently appraise the findings by evaluating all selected studies for quality. However, the evidence continues to grow rapidly. We believe that our search strategy could be useful in performing regular updates of the literature and therefore help evolve the current knowledge.

We are also certain that information retrieved in our reviews will be useful in clarifying the risks for certain population groups, as for example chosen here, and in orienting faster the optimal health management and prevention actions for other potential health crisis should they arise.

We are hopeful that these reviews will provide relevant and timely novel evidence to facilitate public health decisions in the midst of the COVID-19 crisis and identify current gaps in the literature to direct further research.

For peer review only

For peer review only - http://bmjopen.bmj.com/site/about/guidelines.xhtml

Ethics and dissemination

The study will be based on published evidence and is exempt from the ethical approval. The dissemination of findings will include publication in a peer-reviewed journal related to the field and presentation at a scientific conference. In addition, a PHIRI project report will be written.

Acknowledgements

We wish to thank Population Health Information Research Infrastructure (PHIRI) coordination team for initiating and financing this project. We thank documentation experts from Santé Publique France, Laetitia Haroutunian and Sandra Kerzanet, for their valuable advice on formulating the search strategy.

Authors' contributions

LCB, JCo, BD, PJN, NS and MJF identified the research questions. All authors were involved in the conceptualisation of the search strategy.

TM and JG will share the role of a first reviewer, each reviewing half of the retrieved articles; the role of a second reviewer will be divided between LCB, SMB, RH, RFS, FPB, JC, MA, BV, PB, PJN. Acting first and the second reviewer will independently evaluate retrieved records for eligibility and inclusion in the systematic reviews and they will independently extract relevant data. The third-party (JCo), if necessary, will resolve any disagreement between the two reviewers.

TM, JG, JCo, BD and LCB formulated the first version of this article. All authors revised and approved the final version of the article.

Funding statement

These systematic reviews are part of Population Health Information Research Infrastructure (PHIRI) project (available from: https://cordis.europa.eu/project/id/101018317/fr). PHIRI is funded by the European Union's Horizon 2020 research and innovation program under grant agreement No: 101018317.

The protocol was developed in collaboration with all listed institutions. The funding body was not involved in the design of the study; it will not be involved in the collection, analysis and interpretation of data or in writing the manuscript. The content of this publication will represent the views of the authors only and not of the European Commission or any other body of the European Union. The European Commission does not take any responsibility for use that may be made of the information it will contain.

Competing interests statement

Authors declare no competing interests.

For peer review only - http://bmjopen.bmj.com/site/about/guidelines.xhtml

REFERENCES

1. Cucinotta D, Vanelli M. WHO Declares COVID-19 a Pandemic. Acta Biomed. 2020;91(1):157-60.
2. Novel Coronavirus (2019-nCoV) Situation Report – 22 (World Health Organization). 2020 [Available from: https://www.who.int/docs/default-source/coronaviruse/situation-reports/20200211-sitrep-22-ncov.pdf.
3. World Health Organisation (WHO). [Available from: https://www.who.int/emergencies/diseases/novel-coronavirus-2019/situation-reports.
4. Cheng Y, Luo R, Wang K, Zhang M, Wang Z, Dong L, et al. Kidney disease is associated with in-hospital death of patients with COVID-19. Kidney Int. 2020;97(5):829-38.
5. Kumar A, Arora A, Sharma P, Anikhindi SA, Bansal N, Singla V, et al. Is diabetes mellitus associated with mortality and severity of COVID-19? A meta-analysis. Diabetes Metab Syndr. 2020;14(4):535-45.
6. Grasselli G, Greco M, Zanella A, Albano G, Antonelli M, Bellani G, et al. Risk Factors Associated With Mortality Among Patients With COVID-19 in Intensive Care Units in Lombardy, Italy. JAMA Intern Med. 2020;180(10):1345-55.
7. Levin AT, Hanage WP, Owusu-Boaitey N, Cochran KB, Walsh SP, Meyerowitz-Katz G. Assessing the age specificity of infection fatality rates for COVID-19: systematic review, meta-analysis, and public policy implications. European Journal of Epidemiology. 2020;35(12):1123-38.
8. Himmels JPW, Borge TC, Brurberg KG, Gravningen KM. COVID-19: COVID-19 and risk factors for hospital admission, severe disease and death Oslo: Norwegian Institute of Public Health; 2021.
9. Johns Hopkins University of Medicine; Coronavirus resource centre. COVID-19 Dashboard by the Center for Systems Science and Engineering (CSSE) at Johns Hopkins University (JHU) [Available from: https://coronavirus.jhu.edu/map.html.
10. Andrews N, Stowe J, Kirsebom F, Toffa S, Rickeard T, Gallagher E, et al. Effectiveness of COVID-19 vaccines against the Omicron (B.1.1.529) variant of concern. medRxiv. 2021:2021.12.14.21267615.
11. Clegg A, Young J, Iliffe S, Rikkert MO, Rockwood K. Frailty in elderly people. Lancet. 2013;381(9868):752-62.
12. van den Akker M, Buntinx F, Knottnerus JA. Comorbidity or multimorbidity: what's in a name? A review of literature. European Journal of General Practice. 1996;2(2):65-70.
13. Fried LP, Tangen CM, Walston J, Newman AB, Hirsch C, Gottdiener J, et al. Frailty in older adults: evidence for a phenotype. J Gerontol A Biol Sci Med Sci. 2001;56(3):M146-56.
14. Rockwood K, Song X, MacKnight C, Bergman H, Hogan DB, McDowell I, et al. A global clinical measure of fitness and frailty in elderly people. Cmaj. 2005;173(5):489-95.
15. Coste J, Valderas JM, Carcaillon-Bentata L. Estimating and characterizing the burden of multimorbidity in the community: A comprehensive multistep analysis of two large nationwide representative surveys in France. PLoS Med. 2021;18(4):e1003584.
16. Iaccarino G, Grassi G, Borghi C, Ferri C, Salvetti M, Volpe M. Age and Multimorbidity Predict Death Among COVID-19 Patients: Results of the SARS-RAS Study of the Italian Society of Hypertension. Hypertension. 2020;76(2):366-72.
17. McQueenie R, Foster HME, Jani BD, Katikireddi SV, Sattar N, Pell JP, et al. Multimorbidity, polypharmacy, and COVID-19 infection within the UK Biobank cohort. PLoS One. 2020;15(8):e0238091.
18. Woolford SJ, D'Angelo S, Curtis EM, Parsons CM, Ward KA, Dennison EM, et al. COVID-19 and associations with frailty and multimorbidity: a prospective analysis of UK Biobank participants. Aging Clin Exp Res. 2020;32(9):1897-905.
19. Zhang XM, Jiao J, Cao J, Huo XP, Zhu C, Wu XJ, et al. Frailty as a predictor of mortality among patients with COVID-19: a systematic review and meta-analysis. BMC Geriatr. 2021;21(1):186.
20. Yang Y, Luo K, Jiang Y, Yu Q, Huang X, Wang J, et al. The Impact of Frailty on COVID-19 Outcomes: A Systematic Review and Meta-analysis of 16 Cohort Studies. J Nutr Health Aging. 2021;25(5):702-9.

For peer review only - http://bmjopen.bmj.com/site/about/guidelines.xhtml

21. Saragih ID, Advani S, Saragih IS, Suarilah I, Susanto I, Lin CJ. Frailty as a mortality predictor in older adults with COVID-19: A systematic review and meta-analysis of cohort studies. Geriatr Nurs. 2021;42(5):983-92.

22. Pranata R, Henrina J, Lim MA, Lawrensia S, Yonas E, Vania R, et al. Clinical frailty scale and mortality in COVID-19: A systematic review and dose-response meta-analysis. Arch Gerontol Geriatr. 2021;93:104324.

23. Maltese G, Corsonello A, Di Rosa M, Soraci L, Vitale C, Corica F, et al. Frailty and COVID-19: A Systematic Scoping Review. J Clin Med. 2020;9(7).

24. Kastora S, Kounidas G, Perrott S, Carter B, Hewitt J, Myint PK. Clinical frailty scale as a point of care prognostic indicator of mortality in COVID-19: a systematic review and meta-analysis. EClinicalMedicine. 2021;36:100896.

25. Cosco TD, Best J, Davis D, Bryden D, Arkill S, van Oppen J, et al. What is the relationship between validated frailty scores and mortality for adults with COVID-19 in acute hospital care? A systematic review. Age Ageing. 2021;50(3):608-16.

26. Dumitrascu F, Branje KE, Hladkowicz ES, Lalu M, McIsaac DI. Association of frailty with outcomes in individuals with COVID-19: A living review and meta-analysis. Journal of the American Geriatrics Society. 2021;69(9):2419-29.

27. Mair FS, Foster HM, Nicholl BI. Multimorbidity and the COVID-19 pandemic - An urgent call to action. J Comorb. 2020;10:2235042x20961676.

28. Mamelund S-E, Shelley-Egan C, Rogeberg O. The association between socioeconomic status and pandemic influenza: Systematic review and meta-analysis. PloS one. 2021;16(9):e0244346.

29. Niedzwiedz CL, O'Donnell CA, Jani BD, Demou E, Ho FK, Celis-Morales C, et al. Ethnic and socioeconomic differences in SARS-CoV-2 infection: prospective cohort study using UK Biobank. BMC medicine. 2020;18:1-14.

30. Nash D, Qasmieh S, Robertson M, Rane M, Zimba R, Kulkarni S, et al. Household factors and the risk of severe COVID-like illness early in the US pandemic. medRxiv. 2020.

31. Himmels JPW, Qureshi SA, Brurberg KG, Gravningen KM. COVID-19: Long-Term Effects of COVID-19. Rapid review. Oslo: Norwegian Institute of Public Health, 2021. 2021.

32. Dumitrascu F, Branje KE, Hladkowicz ES, Lalu M, McIsaac DI. Association of frailty with outcomes in individuals with COVID-19: A living review and meta-analysis. J Am Geriatr Soc. 2021;69(9):2419-29.

33. National Institute for Health Research. PROSPERO; International prospective register of systematic reviews [Available from: https://www.crd.york.ac.uk/PROSPERO/.

34. Shamseer L, Moher D, Clarke M, Ghersi D, Liberati A, Petticrew M, et al. Preferred reporting items for systematic review and meta-analysis protocols (PRISMA-P) 2015: elaboration and explanation. Bmj. 2015;350:g7647.

35. Morgan RL, Whaley P, Thayer KA, Schünemann HJ. Identifying the PECO: A framework for formulating good questions to explore the association of environmental and other exposures with health outcomes. Environ Int. 2018;121(Pt 1):1027-31.

36. Morgan RL, Thayer KA, Bero L, Bruce N, Falck-Ytter Y, Ghersi D, et al. GRADE: Assessing the quality of evidence in environmental and occupational health. Environ Int. 2016;92-93:611-6.

37. Almirall J, Fortin M. The coexistence of terms to describe the presence of multiple concurrent diseases. J Comorb. 2013;3:4-9.

38. Johnston MC, Crilly M, Black C, Prescott GJ, Mercer SW. Defining and measuring multimorbidity: a systematic review of systematic reviews. Eur J Public Health. 2019;29(1):182-9.

39. St Sauver JL, Boyd CM, Grossardt BR, Bobo WV, Rutten LJF, Roger VL, et al. Risk of developing multimorbidity across all ages in an historical cohort study: differences by sex and ethnicity. BMJ open. 2015;5(2):e006413.

40. Gail MH. Population based study. In: Armitage, &, Colton, editors. Encyclopedia of biostatistics: Wiley, Chichester 1998. p. 3450.

41. Lieb R. Population-Based Study. In: Gellman MD, Turner JR, editors. Encyclopedia of Behavioral Medicine. New York, NY: Springer New York; 2013. p. 1507-8.

For peer review only

For peer review only - http://bmjopen.bmj.com/site/about/guidelines.xhtml

42. World Health Organisation (WHO). COVID-19 Global literature on coronavirus disease 2022 [Available from: https://search.bvsalud.org/global-literature-on-novel-coronavirus-2019-ncov/.
43. Lazarus JV, Palayew A, Rasmussen LN, Andersen TH, Nicholson J, Norgaard O. Searching PubMed to Retrieve Publications on the COVID-19 Pandemic: Comparative Analysis of Search Strings. J Med Internet Res. 2020;22(11):e23449.
44. Makovski TT, Schmitz S, Zeegers MP, Stranges S, van den Akker M. Multimorbidity and quality of life: Systematic literature review and meta-analysis. Ageing Res Rev. 2019;53:100903.
45. Ouzzani M, Hammady H, Fedorowicz Z, Elmagarmid A. Rayyan-a web and mobile app for systematic reviews. Syst Rev. 2016;5(1):210.
46. Page MJ, Moher D, Bossuyt PM, Boutron I, Hoffmann TC, Mulrow CD, et al. PRISMA 2020 explanation and elaboration: updated guidance and exemplars for reporting systematic reviews. Bmj. 2021;372:n160.
47. Alderson P, Green S, Higgins JPT, editors. Cochrane Reviewers' Handbook 4.2.2 [updated March 2004]. In: The Cochrane Library, Issue 1, Chichester, UK: John Wiley & Sons, Ltd. 2004. .
48. Centre for Reviews and Dissemination [CRD]. Systematic Reviews: CRD's Guidance for Undertaking Reviews in Healthcare. 3rd edn. . CRD University of York, York Publishing Services Ltd, York, . 2009.
49. Wells GA, Shea B, O'Connell D, Peterson J, Welch V, Losos M, et al. The Newcastle-Ottawa Scale (NOS) for assessing the quality if nonrandomized studies in meta-analyses. Available from: http://www.ohri.ca/programs/clinical_epidemiology/oxford.asp [
50. Herzog R, Álvarez-Pasquin MJ, Díaz C, Del Barrio JL, Estrada JM, Gil Á. Are healthcare workers' intentions to vaccinate related to their knowledge, beliefs and attitudes? a systematic review. BMC Public Health. 2013;13(1):154.
51. Modesti PA, Reboldi G, Cappuccio FP, Agyemang C, Remuzzi G, Rapi S, et al. Panethnic differences in blood pressure in Europe: a systematic review and meta-analysis. PloS one. 2016;11(1):e0147601.
52. Page MJ MJ, Bossuyt PM, Boutron I, Hoffmann TC, Mulrow CD, et al. . The PRISMA 2020 statement: an updated guideline for reporting systematic reviews. . BMJ 2021:372:n71. .
53. Schisterman EF, Cole SR, Platt RW. Overadjustment bias and unnecessary adjustment in epidemiologic studies. Epidemiology. 2009;20(4):488-95.
54. Viechtbauer W. Conducting Meta-Analyses in R with the metafor Package. Journal of Statistical Software. 2010;36(3):1 - 48.
55. Begg CB, Mazumdar M. Operating Characteristics of a Rank Correlation Test for Publication Bias. Biometrics. 1994;50(4):1088-101.
56. Egger M, Davey Smith G, Schneider M, Minder C. Bias in meta-analysis detected by a simple, graphical test. Bmj. 1997;315(7109):629-34.
57. Duval S, Tweedie R. Trim and fill: A simple funnel-plot-based method of testing and adjusting for publication bias in meta-analysis. Biometrics. 2000;56(2):455-63.
58. Higgins JP, Thompson SG. Quantifying heterogeneity in a meta-analysis. Stat Med. 2002;21(11):1539-58.
59. Tuty Kuswardhani RA, Henrina J, Pranata R, Anthonius Lim M, Lawrensia S, Suastika K. Charlson comorbidity index and a composite of poor outcomes in COVID-19 patients: A systematic review and meta-analysis. Diabetes & Metabolic Syndrome: Clinical Research & Reviews. 2020;14(6):2103-9.
60. Vist GE, Arentz-Hansen EH, Vedøy TF, Spilker RACS, Hafstad EV, Giske L. Incidence and severe outcomes from COVID-19 among immigrant and minority ethnic groups and among groups of different socio-economic status: A systematic review. 2021.
61. Wachtler B, Michalski N, Nowossadeck E, Diercke M, Wahrendorf M, Santos-Hövener C, et al. Socioeconomic inequalities and COVID-19–A review of the current international literature. 2020.
62. Khanijahani A, Iezadi S, Gholipour K, Azami-Aghdash S, Naghibi D. A systematic review of racial/ethnic and socioeconomic disparities in COVID-19. International journal for equity in health. 2021;20(1):1-30.

For peer review only - http://bmjopen.bmj.com/site/about/guidelines.xhtml

63. Lewin S, Glenton C, Munthe-Kaas H, Carlsen B, Colvin CJ, Gülmezoglu M, et al. Using qualitative evidence in decision making for health and social interventions: an approach to assess confidence in findings from qualitative evidence syntheses (GRADE-CERQual). PLoS Med. 2015;12(10):e1001895.
64. Tong A, Flemming K, McInnes E, Oliver S, Craig J. Enhancing transparency in reporting the synthesis of qualitative research: ENTREQ. BMC medical research methodology. 2012;12(1):1-8.
65. WHO Regional Office for Europe. Guide to qualitative evidence synthesis: evidence-informed policy-making using research in the EVIPNET framework. 2021.

For peer review only

For peer review only - http://bmjopen.bmj.com/site/about/guidelines.xhtml

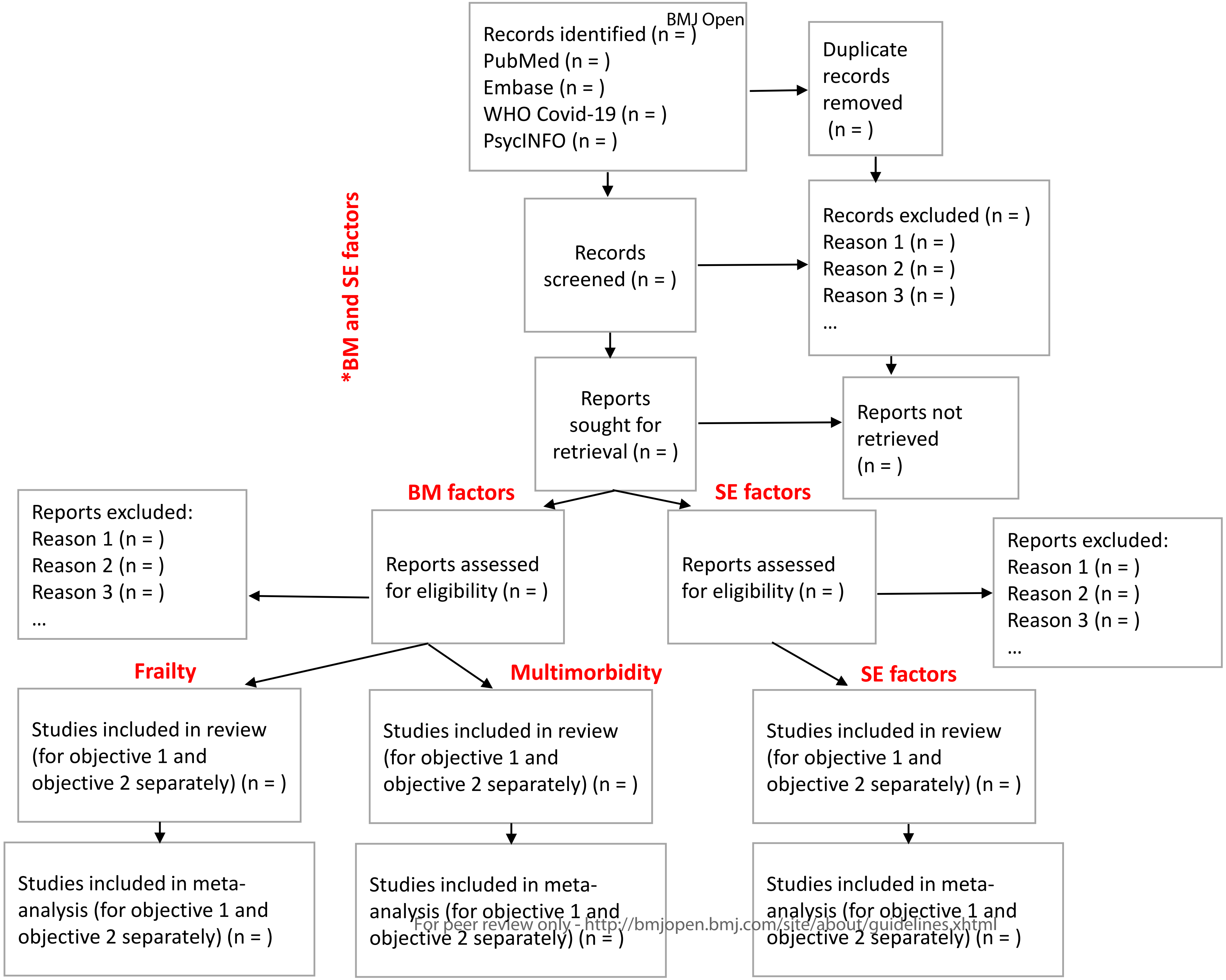

For peer review only - http://bmjopen.bmj.com/site/about/guidelines.xhtml

WHO COVID-19 Global literature on coronavirus disease strategy

((tw:("socio economic" OR "socio-economic" OR socioeconomics OR "socio-economics" OR "socio economics" OR socioeconomic OR "social difference" OR "social differences" OR "social inequality" OR "social inequalities" OR "socioeconomic inequality" OR "socioeconomic inequalities" OR "social disparity" OR "social disparities" OR education OR literacy OR "socioprofessional" OR "socio-professional" OR "socio professional" OR "social conditions" OR "social class" OR "social classes" OR "health status disparity" OR "health status disparities" OR income OR poverty OR deprivation OR rural OR urban OR "housing deprivation" OR homeless OR houseless OR homelessness OR ethnic$ OR race OR emigrant OR immigrant OR migrant OR "minority group" OR "minority groups" OR disadvantaged OR "marital status" OR ((characteristics OR factors OR status) AND (economic OR social OR educational)) OR ((composition OR characteristics OR size) AND (family OR household)))) OR (tw:(multimorbidity OR "multi-morbidity" OR "multi morbidity" OR multimorbidities OR "multi-morbidities" OR "multi morbidities" OR multimorbid OR "multi-morbid" OR "multi morbid" OR comorbidity OR "co-morbidity" OR "co morbidity" OR comorbidities OR "co-morbidities" OR "co morbidities" OR comorbid OR "co morbid" OR "multiple chronic conditions" OR "multiple chronic diseases" OR "multiple conditions" OR "multiple diseases" OR "multiple disorders" OR polymorbid$ OR "poly-morbidity" OR "poly-morbidities" OR "poly morbidity" OR "poly morbidities" OR polypath$ OR pluripath$ OR multipath$ OR "multi pathology" OR "multi pathologies" OR "multi-pathology" OR "multi-pathologies" OR "multiple pathologies" OR "disease cluster" OR "disease clusters" OR "disease pattern" OR "disease patterns" OR "concurrent chronic diseases" OR "multiple chronic disorders")) OR (tw:(frailty OR frail))) AND tw:("cross-sectional" OR "cross sectional" OR "case-control" OR "case control" OR cohort OR longitudinal OR "ecological study" OR "ecological studies" OR "ecological design" OR "ecological designs" OR observational OR "observational study" OR "observational studies" OR "observational design" OR "observational designs" OR "prospective study" OR "prospective studies" OR "prospective design" OR "prospective designs" OR "retrospective study" OR "retrospective studies" OR "retrospective design" OR "retrospective designs" OR "prospective observational study" OR "prospective observational studies" OR "retrospective observational study" OR "retrospective observational studies")

For peer review only

PsycINFO strategy

S1

"cross-sectional" OR "cross sectional" OR "case-control" OR "case control" OR cohort OR longitudinal OR "ecological study" OR "ecological studies" OR "ecological design" OR "ecological designs" OR observational OR "observational study" OR "observational studies" OR "observational design" OR "observational designs" OR "prospective study" OR "prospective studies" OR "prospective design" OR "prospective designs" OR "retrospective study" OR "retrospective studies" OR "retrospective design" OR "retrospective designs" OR "prospective observational study" OR "prospective observational studies" OR "retrospective observational study" OR "retrospective observational studies" OR DE("Longitudinal Studies" OR "Followup studies" OR "Retrospective studies" OR "Cohort analysis")

S2

"socio economic" OR "socio-economic" OR socioeconomics OR "socio-economics" OR "socio economics" OR socioeconomic OR "social difference" OR "social differences" OR "social inequality" OR "social inequalities" OR "socioeconomic inequality" OR "socioeconomic inequalities" OR "social disparity" OR "social disparities" OR education OR literacy OR "socioprofessional" OR "socio-professional" OR "socio professional" OR "social conditions" OR "social class" OR "social classes" OR "health status disparity" OR "health status disparities" OR income OR poverty OR deprivation OR rural OR urban OR "housing deprivation" OR homeless OR houseless OR homelessness OR ethnic* OR race OR emigrant OR immigrant OR migrant OR "minority group" OR "minority groups" OR disadvantaged OR "marital status" OR ((characteristics OR factors OR status) AND (economic OR social OR educational)) OR ((composition OR characteristics OR size) AND (family OR household)) OR DE ("Disadvantaged" OR "Economic Disadvantage" OR "Economic Inequality" OR "Economic Resources" OR "Family Socioeconomic Level" OR "Income (Economic)" OR "Lower Class" OR "Poverty" OR "Rural Health" OR "Socioeconomic Status" OR "Socioeconomic Factors" OR "Social Class" OR "Urban Health" OR "Racial and Ethnic Differences" OR "Racial Disparities" OR "Health Disparities")

S3

multimorbidity OR "multi-morbidity" OR "multi morbidity" OR multimorbidities OR "multi-morbidities" OR "multi morbidities" OR multimorbid OR "multi-morbid" OR "multi morbid" OR comorbidity OR "co-morbidity" OR "co morbidity" OR comorbidities OR "co-morbidities" OR "co morbidities" OR comorbid OR "co morbid" OR "multiple chronic conditions" OR "multiple chronic diseases" OR "multiple conditions" OR "multiple diseases" OR "multiple disorders" OR polymorbid* OR "poly-morbid" OR "poly-morbidity" OR "poly-morbidities" OR "poly morbid" OR "poly morbidity" OR "poly morbidities" OR polypath* OR pluripath* OR multipath* OR "multi pathology" OR "multi pathologies" OR "multi-pathology" OR "multi-pathologies" OR "multiple pathologies" OR "disease cluster" OR "disease clusters" OR "disease pattern" OR "disease patterns" OR "concurrent chronic diseases" OR "multiple chronic disorders" OR Comorbidity [DE]

S4 Frailty OR frail

S5

"covid-19" OR "2019-ncov" OR "sars cov 2" OR "cov-19" OR cov19 OR "cov 19" OR 2019nCoV OR betacoronavirus* OR "corona virus" OR coronavirus* OR coronovirus* OR CoV OR CoV2 OR COVID OR covid19 OR "covid 2019" OR "Severe Acute Respiratory Syndrome Coronavirus 2" OR "2019 ncov" OR (wuhan AND Coronavirus) OR "HCoV-19" OR nCoV OR SARS2 OR SARSCoV OR (sars AND virus) OR "sars virus" OR (sars AND CoV) OR "sars cov" OR "severe acute respiratory syndrome cov" OR "SARS CoV-2" OR "SARS-

For peer review only

PsycINFO strategy

CoV-2" OR SARSCoV2 OR "CoV-2" OR "covid 19" OR covid2019 OR "covid-2019" OR "novel CoV" OR "corona pandemic" OR "wuhan virus" OR "CoV 2" OR ((wuhan OR hubei OR huanan) AND ("severe acute respiratory" OR pneumonia*) AND outbreak*) OR COVID-19 [DE]

S6

(S2 OR S3 OR S4) AND S5 AND S1

For peer review only

Embase strategy

((comorbid* OR 'comorbidity'/exp OR comorbidity OR 'multiple chronic conditions'/exp OR 'multiple chronic conditions' OR multimorbid* OR 'multi morbid' OR 'multi morbidity' OR 'multi morbidities' OR 'co morbid' OR 'co morbidity' OR 'co morbidities' OR 'disease pattern' OR 'disease patterns' OR 'disease cluster' OR 'disease clusters' OR 'multiple conditions' OR 'multiple diseases' OR 'multiple disorders' OR polymorbid* OR 'poly morbid' OR 'poly morbidity' OR 'poly morbidities' OR polypath* OR 'multiple pathology' OR 'multiple pathologies') OR ('frail elderly'/exp OR 'frail elderly' OR 'frailty'/exp OR 'frailty' OR frail*) OR (('socioeconomics'/exp OR 'socioeconomics' OR 'social status'/exp OR 'social status' OR 'social stratification'/exp OR 'social stratification' OR 'social class'/exp OR 'social class') OR ('urban area'/exp OR 'urban area' OR 'rural area'/exp OR 'rural area') OR 'population group'/exp OR ('minority group'/exp OR 'minority group' OR 'homelessness'/exp OR 'homelessness' OR 'migrant'/exp OR 'migrant' OR 'race'/exp OR 'race' OR ethnic* OR 'minority groups') OR ('economic status'/exp OR 'economic status' OR 'income'/exp OR 'income' OR 'income group'/exp OR 'income group') OR ('social discrimination'/exp OR 'social discrimination' OR 'socioeconomic inequality'/exp OR 'socioeconomic inequality' OR 'health disparity'/exp OR 'health disparity' OR 'poverty'/exp OR 'poverty' OR 'income inequality'/exp OR 'income inequality') OR ('educational status'/exp OR 'educational status' OR 'literacy'/exp OR 'literacy' OR 'education* level') OR ('family size'/exp OR 'family size' OR 'household characteristics' OR 'household size' OR 'household'/exp))) AND ('longitudinal study'/exp OR 'longitudinal study' OR 'cross-sectional study'/exp OR 'cross-sectional study' OR longitudinal OR 'cross sectional' OR 'case control study'/exp OR 'case control study' OR 'case control' OR cohort OR 'ecological study' OR 'ecological design' OR 'observational study'/exp OR 'observational study' OR 'retrospective study'/exp OR 'retrospective study' OR 'prospective study'/exp OR 'prospective study' OR 'prospective observational study' OR 'prospective observational studies' OR 'retrospective observational study' OR 'retrospective observational studies' OR 'ecological designs' OR 'prospective studies' OR 'retrospective studies' OR 'observational studies' OR 'ecological studies') AND ('coronavirus disease 2019'/exp OR 'coronavirus disease 2019' OR 'covid 2019' OR 'covid 19' OR covid2019 OR covid19 OR sarscov2 OR 'sars cov 2' OR 'sars cov2' OR 'severe acute respiratory syndrome'/exp OR 'severe acute respiratory syndrome' OR 'wuhan virus' OR 'cov 2' OR cov2 OR coronavirus OR 'corona virus' OR 'novel cov' OR 2019ncov OR '2019 ncov' OR 'corona pandemic' OR betacoronavirus OR cov19 OR 'cov 19' OR 'hcov-19' OR ncov OR sars2) AND [english]/lim AND [2020-2021]/py

For peer review only

PRISMA-S Checklist

Section/topic	#	Checklist item	Location(s) Reported
**INFORMATION SOURCES AND METHODS**			
Database name	1	Name each individual database searched, stating the platform for each.	Page 7 (lines 208,209)
Multi-database searching	2	If databases were searched simultaneously on a single platform, state the name of the platform, listing all of the databases searched.	NA
Study registries	3	List any study registries searched.	NA
Online resources and browsing	4	Describe any online or print source purposefully searched or browsed (e.g., tables of contents, print conference proceedings, web sites), and how this was done.	NA
Citation searching	5	Indicate whether cited references or citing references were examined, and describe any methods used for locating cited/citing references (e.g., browsing reference lists, using a citation index, setting up email alerts for references citing included studies).	Page 8 (216,217)
Contacts	6	Indicate whether additional studies or data were sought by contacting authors, experts, manufacturers, or others.	Page 8 and 9 (217-219)
Other methods	7	Describe any additional information sources or search methods used.	NA
**SEARCH STRATEGIES**			
Full search strategies	8	Include the search strategies for each database and information source, copied and pasted exactly as run.	Page 7 (Table 1) and Annex 1
Limits and restrictions	9	Specify that no limits were used, or describe any limits or restrictions applied to a search (e.g., date or time period, language, study design) and provide justification for their use.	Page 8 (216)
Search filters	10	Indicate whether published search filters were used (as originally designed or modified), and if so, cite the filter(s) used.	NA
Prior work	11	Indicate when search strategies from other literature reviews were adapted or reused for a substantive part or all of the search, citing the previous review(s).	Page 7 and 8 (Table 1)

For peer review only

Updates	12	Report the methods used to update the search(es) (e.g., rerunning searches, email alerts).	Page 9 (219,220)
Dates of searches	13	For each search strategy, provide the date when the last search occurred.	Page 8 (216)
**PEER REVIEW**			
Peer review	14	Describe any search peer review process.	Pages 9, 10, 11
**MANAGING RECORDS**			
Total Records	15	Document the total number of records identified from each database and other information sources.	Page 11 (300,301)
Deduplication	16	Describe the processes and any software used to deduplicate records from multiple database searches and other information sources.	Page 9 (223,224)

PRISMA-S: An Extension to the PRISMA Statement for Reporting Literature Searches in Systematic Reviews
Rethlefsen ML, Kirtley S, Waffenschmidt S, Ayala AP, Moher D, Page MJ, Koffel JB, PRISMA-S Group.
Last updated February 27, 2020.

For peer review only

BMJ Open

Etiologic and prognostic roles of frailty, multimorbidity and socioeconomic characteristics in the development of SARS-CoV-2 health outcomes: protocol for systematic reviews of population-based studies

Journal:	*BMJ Open*
Manuscript ID	bmjopen-2022-063573.R2
Article Type:	Protocol
Date Submitted by the Author:	26-Oct-2022
Complete List of Authors:	Makovski, Tatjana; Sante publique France,<br>Ghattas, Jinane; UCLouvain<br>Monnier Besnard, Stephanie; Sante publique France<br>Ambrozova, Monika; Institute of Health Information and Statistics of the Czech Republic<br>Vasinova, Barbora; Institute of Health Information and Statistics of the Czech Republic<br>Feteira-Santos , Rodrigo ; Universidade de Lisboa, Instituto de Saúde Ambiental, Faculdade de Medicina<br>Bezzegh, Peter; Government of Hungary<br>Ponce Bollmann, Felipe; Universidad Nacional de Educación a Distancia<br>Cottam, James; Sciensano<br>Haneef, Romana; Sante publique France<br>Devleesschauwer, Brecht; Sciensano, Epidemiology and public health<br>Speybroeck, Niko; Université catholique de Louvain, Institut de recherche santé et société (IRSS)<br>Nogueira, Paulo; Universidade de Lisboa Faculdade de Medicina<br>Forjaz, Maria João; National School of Public Health and Health Service Research Network for Chronic Diseases (Red de Investigación en Servicios de Salud en Enfermedades Crónicas/REDISSEC), Carlos III Institute of Health.<br>Coste, Joel; Sante publique France<br>Carcaillon-Bentata, Laure; Sante publique France
**Primary Subject Heading**:	Epidemiology
Secondary Subject Heading:	Public health
Keywords:	COVID-19, EPIDEMIOLOGY, PUBLIC HEALTH

SCHOLARONE™ Manuscripts

Etiologic and prognostic roles of frailty, multimorbidity and socioeconomic characteristics in the development of SARS-CoV-2 health outcomes: protocol for systematic reviews of population-based studies

Authors

Tatjana T. Makovski[1], Jinane Ghattas[2], Stéphanie Monnier-Besnard[1], Monika Ambrožová[3], Barbora Vašinová[3], Rodrigo Feteira-Santos[4,11], Peter Bezzegh[5], Felipe Ponce Bollmann[6], James Cottam[7,10], Romana Haneef[1], Brecht Devleesschauwer[7,9], Niko Speybroeck[2], Paulo Jorge Nogueira[4,11], Maria João Forjaz[8], Joël Coste[1], Laure Carcaillon-Bentata[1]

Authors' affiliations

[1] Public health France, France

[2] Institute of Health and Society, Université catholique de Louvain, Belgium

[3]Institute of Health Information and Statistics of the Czech Republic, Czech Republic

[4]Área Disciplinar Autónoma de Bioestatística, Faculdade de Medicina, Universidade de Lisboa, Lisboa, Portugal

[5]Directorate for Project Management, National Directorate General for Hospitals , Hungary

[6]Universidad Nacional de Educación a Distancia, Spain

[7]Department of Epidemiology and Public Health, Sciensano, Brussels, Belgium

[8]National Center of Epidemiology, Instituto de Salud Carlos III, REDISSEC and RICAPPS, Spain

[9]Department of Translational Physiology, Infectiology and Public Health, Ghent University, Merelbeke, Belgium

[10]Institute of Tropical Medicine, Antwerp, Belgium

[11]Instituto de Saúde Ambiental, Faculdade de Medicina, Universidade de Lisboa, Lisboa, Portugal

Corresponding author

Tatjana T. Makovski

Agence nationale de santé publique
12 rue du Val d'Osne
94415 Saint-Maurice Cedex France
www.santepubliquefrance.fr

tatjanamakovski@hotmail.com

jinane.ghattas@uclouvain.be; stephanie.monnierbesnard@santepubliquefrance.fr; monika.ambrozova@uzis.cz; Barbora.Vasinova@uzis.cz; rodrigosantos@medicina.ulisboa.pt; bezzegh.peter@okfo.gov.hu; fponce22@alumno.uned.es; James.Cottam@sciensano.be; Romana.HANEEF@santepubliquefrance.fr; Brecht.Devleesschauwer@sciensano.be; niko.speybroeck@uclouvain.be; paulo.nogueira@edu.ulisboa.pt; jforjaz@isciii.es; Joel.COSTE@santepubliquefrance.fr; Laure.CARCAILLON-BENTATA@santepubliquefrance.fr

For peer review only

Abstract

Introduction: There is growing evidence that the impact of COVID-19 crisis may be stronger for individuals with multimorbidity, frailty and lower socioeconomic status. Existing reviews focus on few, mainly short-term effects of COVID-19 illness and patients with single chronic disease. Information is also largely missing for population representative samples.

Applying population-based approach, the systematic reviews will have two objectives, to: 1) evaluate the etiologic roles of frailty, multimorbidity and socioeconomic status on SARS-CoV-2 infection probability, hospitalization, intensive care unit (ICU) admission, mechanical ventilation and COVID-19 related mortality among general population; 2) investigate the prognostic roles of frailty, multimorbidity and socioeconomic characteristics on the risk of hospitalization, ICU admission, mechanical ventilation, COVID-19 mortality, functioning, quality of life, disability, mental health and work absence.

Methods and analysis: For this ongoing work, four databases were searched: PubMed, Embase, WHO COVID-19 Global literature on coronavirus disease and PsycINFO, for the period between January 2020 and April 7 2021. Peer-reviewed published literature in English will be considered and all types of population-based studies. Studies using standard tools to assess multimorbidity such as disease count, comorbidity indices or disease combinations will be retained, as well as studies with standard scales and scores for frailty or measurement of a socioeconomic gradient. Initial search included 10,139 articles, 411 for full-text reading. Results will be summarized by risk factor, objective and outcome. The feasibility of meta-analysis will be determined by the findings and will aim to better understand uncertainties of the results. Quality of studies will be assessed using standardized scales.

Ethics and dissemination: The study will be based on published evidence and it is exempt from the ethical approval. This work is part of the Population Health Information Research Infrastructure (PHIRI) project. Dissemination of the results will imply conference presentation, submission for scientific publication and PHIRI project report.

Prospero registration: CRD42021249444.

For peer review only

Strengths and limitations of this study

- This Prisma guided protocol describes methodology of systematic reviews regarding the roles of multimorbidity, frailty and socioeconomic risk factors in development of SARS-CoV-2 health outcomes
- Only quantitative studies conducted on population representative samples will be eligible
- Only peer-reviewed literature published in English will be considered
- The literature screening process, data extraction and study quality assessment will be performed in pairs
- The reviews may be limited with the heterogeneity of available studies in terms of diversity of measurement tools used to assess risk factors and outcomes

For peer review only

Introduction

The early months of 2020 were marked with rapid growth in infections caused by the novel severe acute respiratory syndrome coronavirus 2 or SARS-CoV-2, leading to the state of a global pandemic declared by the World Health Organization (WHO) on March 11, 2020 (1). SARS-CoV-2 causes coronavirus disease 2019 (COVID-19) (2), which has led to severe health and health care management consequences around the world (3).

It has been demonstrated so far that age, male sex and comorbidity are the main risk factors for COVID-19 hospitalisations, intensive care unit (ICU) admissions and mortality (4-6). Specifically, infection fatality rates due to COVID-19 have been found to exponentially increase with age : 0.4% at age 55, 1.4% at age 65, 4.6% at age 75 and 15% at age 85 (7). In addition, having at least one comorbidity more than doubled the risk of hospitalisation while certain comorbidities, including severe kidney disease, diabetes, severe immunodeficiency or heart failure, among few others, increased this risk in particular, e.g., odds ratio (OR)/hazard ratio (HR) ≥ 3 for hospitalisation or death (8). Much remains to be clarified due to the lifespan of the new disease of only two years and the appearance of new virus variants (9) against which the same protection level conferred by existing vaccines is not guaranteed (10). In particular, regarding which and why certain population groups still display more severe symptoms and outcomes compared to others.

Frailty, as a marker of increased vulnerability due to accumulated deficits in multiple body systems (11) and multimorbidity, commonly defined as having two or more chronic conditions (12) are well recognized risk factors for adverse health outcomes in the elderly population, including hospitalisation, dependency and mortality (13-15). In the context of the COVID-19 pandemic, it is plausible that both frailty and multimorbidity increase the risk of SARS-CoV-2 infection and the development of severe outcomes by altering older people's biological background and immunity. Some studies have found that frailty and multimorbidity predicted COVID-19 patients' prognosis for hospitalisation, mortality and level of care at discharge (16-26). To date, however, most studies addressing these relationships are likely to be biased due to the inclusion of only severe COVID-19 cases admitted to the hospital. Whether the relationships of frailty and multimorbidity with COVID-19 infection and outcomes hold for non-severe forms of COVID-19, it is of major interest to improve risk stratification and public health guidance in the general population (27).

Genetic and biomedical factors are not the only determinants that may influence COVID-19 outcomes. During previous pandemics, lower socioeconomic status was associated with a higher disease burden. It is therefore important to consider social vulnerability (28). Several studies suggest that COVID-19, like other infectious diseases, disproportionally affects socially disadvantaged population groups and places them at a higher risk of disease severity. People of low socioeconomic status, ethnic minority groups (29) and crowded households (30) had higher risks of acquiring COVID-19 and higher hospitalisation rates. Overall, a large number of studies with very different methodological approaches (settings, type of adjustment) have been conducted to assess the role of socioeconomic factors in SARS-CoV-2 infection and outcomes. Therefore, it is important to synthesize the current knowledge from well-designed population-based studies in order to clarify and quantify the weight of each socioeconomic factor.

For peer review only

Finally, most of the investigated COVID-19 related health impacts appear so far to be of short-term, e.g. hospitalisations, ICU admissions or mortality. Evidence on long-term effects such as potential mental health difficulties, impact on quality of life or functioning, among others, in COVID-19 patients is limited (31, 32), but it will certainly grow as the pandemic prolongs.

The above assumptions justify the relevance, need and importance of summarizing current knowledge using a systematic review approach regarding the etiologic and prognostic roles of frailty, multimorbidity and socioeconomic characteristics in developing COVID-19 disease and related short and long-term health consequences, among general population. The Prospero registry for systematic reviews (33) was cross-checked to ensure the novelty of the research questions.

Objectives

This paper aims to describe the methodology, which will be used for conducting systematic reviews intended at synthesising knowledge on the roles of frailty, multimorbidity and socioeconomic characteristics in the acquisition and evolution of COVID-19 related health outcomes. Two main study objectives have been determined.

The first objective is to explore the effects of frailty, multimorbidity and socioeconomic characteristics on SARS-CoV-2 infection risk in the general population. The goal is to consider the general population at risk of developing SARS-CoV-2 infection or related outcomes and to estimate a potential increase in risk associated with a given risk factor (frailty or multimorbidity or socioeconomic characteristics). The outcomes of interest are infection by SARS-CoV-2, hospitalisation for COVID-19, ICU admission, mechanical ventilation and mortality due to COVID-19.

The second objective is to explore the prognostic effects of frailty, multimorbidity and socioeconomic characteristics in SARS-CoV-2 infected subjects from the general population. The goal is to consider SARS-CoV-2 infected individuals from the general population and estimate a potential increase in risk of developing severe short- and long-term outcomes associated with a given risk factor (frailty, multimorbidity or socioeconomic characteristics). The outcomes of interest are death due to COVID-19, hospitalization, ICU admission, mechanical ventilation, functioning, quality of life, disability, mental health difficulties and work absence/sick leave.

Methods and analysis

Protocol and registration

This protocol is written along the requirements of Preferred Reporting Items for Systematic Review and Meta-Analysis Protocols (PRISMA-P) 2015 checklist (34). The study is registered in the Prospero registry for systematic review protocols under the registration number: CRD42021249444.

Should there be any substantial amendments to this protocol in the course of the study, they will be fully documented and included in dissemination, such as reports and scientific publications.

Eligibility criteria

We applied the Population, Exposure, Comparator and Outcomes (PECO) framework (35) to clearly specify research objectives. This framework has been increasingly recognized in population health, environmental and

For peer review only

occupational health fields due to the importance of identifying the exposure and clarifying its association with the outcome using comparator groups; (35, 36). The study objectives are hence further detailed as follows:

Objective 1

P Population = general or well-defined population SARS-CoV-2 negative

E Exposure = (either) frailty, multimorbidity, lower socioeconomic status

C Comparator = general population with no frailty or no multimorbidity or with better socioeconomic status

O Outcome(s) = infection by SARS-COV-2, hospitalization for COVID-19, ICU admission, mechanical ventilation, mortality due to COVID-19

Objective 2

P Population = general or well-defined population SARS-CoV-2 infected (COVID-19 diagnosed, with e.g. polymerase chain reaction (PCR) test, medical imaging or similar)

E Exposure = (either) frailty, multimorbidity, lower socioeconomic status

C Comparator = SARS-CoV-2 infected subjects from the population with no frailty or no multimorbidity or with better socioeconomic status

O Outcome(s) = death due to COVID-19, hospitalization for COVID-19, ICU admission, mechanical ventilation, functioning, quality of life, disability, mental health difficulties and work absence/sick leave.

A widely agreed definition of multimorbidity is lacking in the scientific literature (37, 38), however the most frequently cited definition is a coexistence of two or more conditions within an individual (12, 38) which will likewise apply in this review. Multimorbidity can also be operationalised differently, in a sense that it is commonly explored as a disease count (≥ 2, ≥ 3 diseases or similar), through comorbidity/multimorbidity indices or disease combinations. To summarize all relevant evidence, studies with any of the valid mentioned measurement tools that observe the association between minimum of two conditions and COVID-19 related outcomes will be retained. In the final summary table, this review will specify the multimorbidity measurement tool, and the number and list of conditions for each included study.

Similarly, number of definitions for frailty can be found in the literature where each holds its advantages and limitations. Two main approaches coexist and are widely used to define frailty: the phenotypic approach described by Fried et al. in 2001 (13) and the deficit accumulation approach proposed by Rockwood et al. in 2004 (14). We choose to include all studies using a definition of frailty based on one of these two approaches, provided that its predictive value had been previously validated. Studies using the term "frailty" without reference to either of these two standards will not be included.

Likewise, literature looking at the role of the socioeconomic gradient in COVID-19 related outcomes will be retained. Socioeconomic status assessment will include standard indicators, such as income, education, occupation, employment, housing, urban/rural setting, household size, race and marital status measured at the individual or the community level (ecological studies). All age groups will be taken into account considering that socioeconomic differences may be observed across all ages and that multimorbidity is increasingly present among younger individuals as well as among older adults (39).

For peer review only

Eligible studies and settings

To identify population based studies, the following definitions will apply: “a population-based study is defined as a study of properties of a well-defined population, such as individuals residing in a defined geographic region in a given time period”(40) and “population-based studies aim to answer research questions for defined populations; answers should be generalizable to the whole population addressed in the study hypothesis, not only to the individuals included in the study” (41). Therefore, the following examples of population-based studies may be eligible for our reviews: community-dwelling, hospital-based, nursing homes, homes for elderly, or similar, as long as the population in the study is representative of the country or region from which the sample was drawn. Only quantitative studies will be considered.

Study designs

All types of etiologic and prognostic observational studies with comparator groups will be eligible, such as cohorts, cross-sectional studies, case-control studies or ecological studies (for socioeconomic determinants only). Interventions and clinical trials will be excluded, as well as qualitative and case studies. Only published peer-reviewed original studies written in English will be considered.

Information sources and search strategy

The following databases were explored: PubMed, Embase, WHO COVID-19 Global literature on coronavirus disease (42) and PsycINFO. The search strategy considers all possible variations of the terms: multimorbidity, frailty, terms defining socioeconomic characteristics, COVID-19 and study design types. The search strategy was adjusted to the technical specificities of each database. The example of the search strategy in PubMed is presented in Table 1. The search strategy for remaining three databases is presented in Annex 1.

Table 1 PubMed search strategy

#1	Covid-19 (Adjusted from Lazarus et al.)(43)	((((((((((((((((((((("Betacoronavirus"[MeSH Terms] OR "Coronavirus Infections"[MeSH Terms]) OR "COVID-19"[Supplementary Concept]) OR "Coronavirus"[MeSH Terms]) OR "Severe Acute Respiratory Syndrome Coronavirus 2"[Supplementary Concept]) OR "2019nCoV"[All Fields]) OR "betacoronavirus*"[All Fields]) OR "corona virus*"[All Fields]) OR "coronavirus*"[All Fields]) OR "coronovirus*"[All Fields]) OR "CoV"[All Fields]) OR "CoV2"[All Fields]) OR "COVID"[All Fields]) OR (("COVID-19"[Supplementary Concept] OR "COVID-19"[All Fields]) OR "covid19"[All Fields])) OR (((((("COVID-19"[All Fields] OR "covid 2019"[All Fields]) OR "Severe Acute Respiratory Syndrome Coronavirus 2"[Supplementary Concept]) OR "Severe Acute Respiratory Syndrome Coronavirus 2"[All Fields]) OR "2019 ncov"[All Fields]) OR "SARS CoV 2"[All Fields]) OR "2019nCoV"[All Fields]) OR (("wuhan"[All Fields] AND ("Coronavirus"[MeSH Terms] OR "Coronavirus"[All Fields])) AND (2019/12/1:2019/12/31[Date - Publication] OR 2020/1/1:2020/12/31[Date - Publication])))) OR "HCoV-19"[All Fields]) OR "nCoV"[All Fields]) OR "SARS CoV 2"[All Fields]) OR "SARS2"[All Fields]) OR "SARSCoV"[All Fields]) OR (((("sars virus"[MeSH Terms] OR ("sars"[All Fields] AND "virus"[All Fields])) OR "sars virus"[All Fields]) OR ("sars"[All Fields] AND "CoV"[All Fields])) OR "sars cov"[All Fields])) OR (("Severe Acute Respiratory Syndrome Coronavirus 2"[Supplementary Concept] OR "Severe Acute Respiratory Syndrome Coronavirus 2"[All Fields]) OR "SARS CoV 2"[All Fields])) OR "severe acute respiratory syndrome cov*"[All Fields]) AND

For peer review only

		(2019/11/17:3000/12/31[Date - Entry] OR 2019/11/17:3000/12/31[Date - Publication]) OR "COVID-19"[MeSH Terms] OR "SARS-Cov-2"[MeSH Terms] OR "SARS CoV-2" OR "SARS-CoV-2" OR SARSCoV2 OR "CoV-2" OR "covid 19" OR covid2019 OR "covid-2019" OR "novel CoV" OR "corona pandemic*" OR "wuhan virus*" OR "CoV 2" OR ((wuhan OR hubei OR huanan) AND ("severe acute respiratory" OR pneumonia*) AND outbreak*)
#2	Frailty	frailty OR frail OR frailty[MeSH Terms]
#3	Multimorbidity (Adjusted from Makovski et al.)(44)	multimorbidity OR "multi-morbidity" OR "multi morbidity" OR multimorbidities OR "multi-morbidities" OR "multi morbidities" OR multimorbid OR "multi-morbid" OR "multi morbid" OR comorbidity OR "co-morbidity" OR "co morbidity" OR comorbidities OR "co-morbidities" OR "co morbidities" OR comorbid OR "co morbid" OR "multiple chronic conditions" OR "multiple chronic diseases" OR "multiple conditions" OR "multiple diseases" OR "multiple disorders" OR polymorbid* OR "poly-morbid*" OR "poly morbid*" OR polypath* OR pluripath* OR multipath* OR "multi path*" OR "multi-path*" OR "multiple pathologies" OR "disease cluster" OR "disease clusters" OR "disease pattern" OR "disease patterns" OR "concurrent chronic diseases" OR "multiple chronic disorders" OR multimorbidity[MeSH Terms] OR comorbidity[MeSH Terms]
#4	Socioeconomic characteristics	"socio economic" OR "socio-economic" OR socioeconomics OR "socio-economics" OR "socio economics" OR socioeconomic OR "social difference" OR "social differences" OR "social inequality" OR "social inequalities" OR "socioeconomic inequality" OR "socioeconomic inequalities" OR "social disparity" OR "social disparities" OR education OR literacy OR "socioprofessional" OR "socio-professional" OR "socio professional" OR "social conditions" OR "social class" OR "social classes" OR "social class"[MeSH Terms] OR "socioeconomic factors"[MeSH Terms] OR health status disparities[MeSH Terms] OR income OR poverty OR deprivation OR rural OR urban OR "housing deprivation" OR homeless OR houseless OR homelessness OR ethnic* OR race OR emigrant OR immigrant OR migrant OR "minority group" OR "minority groups" OR disadvantaged OR "marital status" OR ((characteristics OR factors OR status) AND (economic OR social OR educational)) OR ((composition OR characteristics OR size) AND (family OR household))
#5	Study design	"cross-sectional" OR "cross sectional" OR "case-control" OR "case control" OR cohort OR longitudinal OR "ecological study" OR "ecological studies" OR "ecological design" OR "ecological designs" OR observational OR "observational study" OR "observational studies" OR "observational design" OR "observational designs" OR "prospective study" OR "prospective studies" OR "prospective design" OR "prospective designs" OR "retrospective study" OR "retrospective studies" OR "retrospective design" OR "retrospective designs" OR "prospective observational study" OR "prospective observational studies" OR "retrospective observational study" OR "retrospective observational studies" OR case-control studies[MeSH Terms] OR cohort studies[MeSH Terms] OR cross-sectional studies[MeSH Terms]
#6		#2 OR #3 OR #4
#7		#1 AND #5 AND #6

The search was restricted using date (January 2020 to April 7, 2021) and language (English) filters. Reference lists of retained studies will be explored for potentially omitted relevant records. For studies where pertinent data are not presented but seem to be available or if measurements of interest (SARS-CoV-2 diagnosis, outcomes and

For peer review only

For peer review only - http://bmjopen.bmj.com/site/about/guidelines.xhtml

risk factors) are not clearly defined, study authors will be contacted for details. If deemed relevant, update of the literature search will be performed.

Study records

Data management

The literature will be managed using Rayyan (45), EndNote and Excel. Records retrieved from the four databases will be exported in EndNote for deduplication. Title and abstract screening will be performed in Rayyan, a web and mobile app for systematic reviews. Full-text articles will be read in EndNote and data will be extracted in customised Excel tables. Two researchers will perform the screening process and data extraction. Considering the actuality of the topic, the quantity of the literature envisaged and the need for timely information, several researchers will be involved in the screening process. For example, two researchers will assume the role of the first reviewer while several colleagues will act as the second reviewer. Weekly meetings will ensure a clear understanding of the study process. In addition, prior to the start of the study selection, a pilot test is foreseen; for instance, homogeneity in the understanding of the exclusion and inclusion criteria among reviewers will be compared and explained on a minimum of 50 studies.

Selection process

Applying the exclusion criteria displayed in List 1, two reviewers will independently screen all records by title and abstract (records screening phase) to select studies for full-text reading (reports screening phase) (46).

List 1: Reasons for exclusion from the systematic review (title/abstract or records screening phase)

1 = Language other than English

2 = Not an original research (e.g. editorial, protocol, etc.) or no original results

3 = Unrelated topic

4 = Not a population-based study

5 = Subpopulation (medical staff, students, pregnant women, etc.)

6 = Duplicate

The criteria above will be used in a hierarchical order to reduce the discrepancy in selected exclusion criteria between the reviewers, implying that should there be more than one criterion applicable to a study, the one higher on the list will be assigned.

Exclusion criteria in List 2 will apply for selecting studies during a full-text (reports) reading phase.

List 2: Reasons for exclusion from the systematic review (full-text reading or reports screening phase)

1 = Not a population-based study

2 = SARS-CoV-2 infection diagnosis not clear*

For peer review only

3 = Study does not consider people with frailty or multimorbidity OR does not contain information on socioeconomic characteristics

4 = Outcome not within the scope of our objectives

5 = Outcome measurement tool not clear*

6 = Risk factor measurement tool not clear*

7 = Subpopulation (e.g. pregnant women, health care workers, students etc.)

8 = Not an original research (e.g. editorial, protocol, review, conference abstract, grey literature, etc.), no original results or not peer-reviewed

9 = The same or largely the same population already considered in another study for the same outcome

10 = Clinical trial or intervention study

11 = Qualitative study

12 = Descriptive study, absence of a comparator group and/or no measure of the association of interest

13 = Other (explain)

*even after contacting authors

The records will be rated as eligible/not eligible/questionable (47) at both screening stages. Any disagreement between the reviewers will be resolved by consensus in referral to this protocol; a third reviewer will be consulted if necessary. Disagreement between the reviewers will be reported in percentages (48).

During the screening process, all articles will be sorted by type of exposure factors, e.g. frailty and multimorbidity on one side (as biomedical determinants) and socioeconomic characteristics on the other, and by objective (objective 1 or 2). Articles on biomedical determinants will later be divided to studies on frailty or multimorbidity. Retained studies will be kept for further analyses.

Data items

Six extraction tables for each exposure and objective will be prepared. They will include: study title, authors, year of publication, study design, study setting and duration, population, sample size, COVID-19 diagnostic criteria, risk factors and study outcomes and means by which they were assessed, measurement of the association between variables of interest, and study adjustment factors.

Two reviewers will extract data independently and compare for agreement (48).

Risk of bias in individual studies

Two reviewers will separately assess a risk of bias for each study retained for final reporting. Any disagreement will be resolved by consensus; otherwise, a third reviewer will be consulted. The Newcastle-Ottawa scales will be used to assess the quality of case-control and cohort studies (49). Each of these scales contains eight items grouped in three categories: selection, comparability and outcome or exposure (for cohort and case-control studies, respectively). Selection items refer to e.g. representativeness of the sample, ascertainment of the exposure, definitions of case and control group, etc. Comparability items inquire on comparability between e.g. cases and controls or between cohorts based on the study design or analysis. Outcomes category items account for the assessment of the outcome or adequacy of the follow up, while exposure items account for e.g.

For peer review only

ascertainment of the exposure or response rate. Each scale item can carry a maximum of one point, except for the comparability group, where up to two points for an item can be assigned, totalling a maximum of nine points for study quality. Higher score indicates higher study quality.

An adjusted version of the Newcastle-Ottawa scale will be used to assess quality for cross-sectional studies (50, 51). Here maximum of 10 points can be assigned.

Abovementioned scales will also apply to assess study quality of ecological studies (for socioeconomic risk factor only), depending of the ecological study design.

Data synthesis

The literature review is ongoing. The search included 10,139 articles in title and abstract screening phase, of which 411 were retained for full-text reading. The screening process results will be presented with PRISMA diagram (52). Given the interest in several risk factors, the PRISMA diagram may be adjusted, as suggested in Fig. 1.

Fig. 1 PRISMA 2020 flow diagram for new systematic reviews, which included searches of databases and registers only (adjusted)

**BM = Biomedical, SE= Socioeconomic*

The final set of retained studies will be described using standardised tables. They will be summarised within each objective, by type of risk factor and study outcome and presented in tabulated form. Study characteristics will be described as well as their main findings.

When possible, meta-analysis models will be performed by each objective, for each risk factor and for a specific outcome with the aim to better understand the uncertainties (random error and bias) of the obtained results. The feasibility of performing meta-analyses will depend on additional criteria, such as number of studies applying the same measurement tools for risk factors and evaluating the same outcomes, as well as reporting odds ratio, hazard ratio, risk ratio or slope. For the meta-analysis, a minimum of four studies will be required. In studies assessing the role of socioeconomic inequalities on health outcomes, unadjusted studies stratified by age group will be sought to avoid overadjustment (53). For studies assessing the roles of biomedical factors, adjusted analyses will be preferred. Adjustment factors will be thoroughly reported. Random effects meta-analyses will be performed with R software using the metafor package (54). Findings will be presented with forest plot(s). Publication bias will be visually assessed using funnel plots and formally tested using Begg and Mazumdar's rank correlation test (55) and Egger's regression test (56). When significant publication bias will be present, Duval and Tweedie's trim-and-fill method will be used to explore the impact on model estimates (57). Heterogeneity will be explored by computing I^2 statistic (58). When feasible, sensitivity analysis or meta-regression using study quality as a covariate will be conducted. Other covariates such as proper operationalisation of multimorbidity or frailty (e.g. number and list of diseases clearly provided or proper definition used), mean age or gender proportion, may also be considered.

For peer review only - http://bmjopen.bmj.com/site/about/guidelines.xhtml

Patient and public involvement

Patients and/or the public were not involved in the design of the protocol and will not be involved in conducting the study, neither in reporting or dissemination plans of the research.

Discussion

This protocol aims to present the methodology that will be applied to systematically assess the evidence on the etiologic and prognostic roles of multimorbidity, frailty and socioeconomic risk factors in the development of SARS-CoV-2 infection and COVID-19 related severe short- and long-term health outcomes, among the general population. These reviews should inform on the quantity and quality of the relevant literature and summarise the existing evidence. In addition, the reviews will report which were the preferred assessment methods for the risk factors and most frequent study outcomes considered, as well as which were the statistical approaches and adjustment factors across studies.

To date, no systematic review on the link between multimorbidity and SARS-CoV-2 infection and outcomes has been identified. Even tough not specifically focusing on patients with multimorbidity, Kuswardhani et al. (59) have summarised evidence on the association between the Charlson's score and poor short-term outcomes in COVID-19 patients. This score is still frequently used to assess health status in patients with multiple conditions, in the absence of a more specific and widely accepted multimorbidity tool. We plan to elaborate on other multimorbidity measurements and broader COVID-19 related consequences, and investigate the possible etiologic role of multimorbidity in the illness acquisition and development. In the context of ageing societies where an increasing number of people live with multiple conditions, scarcity of evidence and public health advice for this population group in the COVID-19 crisis is not acceptable. It is essential to inform how to best evaluate the risks for people with multiple number and combinations of diseases to provide clinicians and policy makers with adequate advice for the most optimal and evidence-based decisions (27).

On the other hand, several systematic reviews have been conducted among frail population (22, 25). However, these focus on hospital settings, usually single centre or few multicentre settings and short-term outcomes; prognostic roles of frailty are also mainly explored. None of the identified reviews investigated the role of frailty in the development of COVID-19 related outcomes in population representative samples. Our systematic review intends to build on the existing evidence by examining the occurrence of a wide number of COVID-19 health outcomes accounting for etiologic as well as prognostic roles of frailty, among the general population.

Similarly, reviews show existing disparities in COVID-19 outcomes in lower socioeconomic groups and ethnic minorities. Specific contexts such as low level of education, poor housing conditions and crowded households could play a role in worsening COVID-19 outcomes (60-62). As above, no systematic review to date has investigated COVID-19 outcomes and severity related to socioeconomic gradient in well-defined population based studies. We, therefore aim to fill this gap. Whereas the three types of factors (multimorbidity, frailty and socioeconomic factors) will be considered and analyzed separately, the similar use of a population-based approach justify the definition of a common methodology and the sharing of the first steps of the reviews performed.

For peer review only

A first limitation of these systematic reviews is the focus on quantitative studies, using comparator groups and providing measures of associations between considered exposures and outcomes. Case and qualitative studies, which may be informative of more complex connections between multimorbidity, frailty, socioeconomic status, and COVID-19, but whose evidence synthesis require the use of methodologies different from systematic reviews of quantitative studies (63-65), will be left aside for the sake of feasibility. A second limitation of this review more specifically concerns socioeconomic status indicators which are often relational and contextual (to a country, a region, a city) and that heterogeneity may result from their use, precluding meta-analyses in several cases. A third potential limitation of the reviews is that only publications written in English will be considered. With this, we possibly risk to overlook national population representative studies, which may have been in the necessity of the crisis as well as for local purposes, released in local languages exclusively. On the other hand, a publication in peer-reviewed international journals in English may also be regarded as an additional study quality check. A strength and a limitation could be the number of scientists who are planned for this study. Involving several team members may accelerate the review process, which is desired when information is crucial. However, one needs to consider the heterogeneity among reviewers in understanding the study method and the review criteria. We will overcome this by conducting a pilot test and holding regular team and individual meetings with involved parties. Moreover, the two first reviewers, experts in respectful areas, will remain the same throughout the screening and data extraction process. Accurately following validated guidelines in preparing this protocol, conducting and reporting the study will help to limit any possible biases to a minimum.

It is worth to mention a high quantity and speed of which the literature is produced during the COVID-19 crisis, as well as a high pace at which data become obsolete. Our reviews intend to provide some of the first estimates of the association between the chosen risk factors and various COVID-19 related outcomes at the population level, by compiling evidence published in a limited timeframe. We will critically and prudently appraise the findings by evaluating all selected studies for quality. However, the evidence continues to grow rapidly. We believe that our search strategy could be useful in performing regular updates of the literature and therefore help evolve the current knowledge.

We are also certain that information retrieved in our reviews will be useful in clarifying the risks for certain population groups, as for example chosen here, and in orienting faster the optimal health management and prevention actions for other potential health crises should they arise.

We are hopeful that these reviews will provide relevant and timely novel evidence to facilitate public health decisions in the midst of the COVID-19 crisis and identify current gaps in the literature to direct further research.

For peer review only

Ethics and dissemination

The study will be based on published evidence and is exempt from the ethical approval. The dissemination of findings will include publication in a peer-reviewed journal related to the field and presentation at a scientific conference. In addition, a PHIRI project report will be written.

Acknowledgements

We wish to thank Population Health Information Research Infrastructure (PHIRI) coordination team for initiating and financing this project. We thank documentation experts from Santé Publique France, Laetitia Haroutunian and Sandra Kerzanet, for their valuable advice on formulating the search strategy.

Authors' contributions

LCB, JCo, BD, PJN, NS and MJF identified the research questions. All authors were involved in the conceptualisation of the search strategy.

TM and JG will share the role of a first reviewer, each reviewing half of the retrieved articles; the role of a second reviewer will be divided between LCB, SMB, RH, RFS, FPB, JC, MA, BV, PB, PJN**.** Acting first and the second reviewer will independently evaluate retrieved records for eligibility and inclusion in the systematic reviews and they will independently extract relevant data. The third-party (JCo), if necessary, will resolve any disagreement between the two reviewers.

TM, JG, JCo, BD and LCB formulated the first version of this article. All authors revised and approved the final version of the article.

Funding statement

These systematic reviews are part of Population Health Information Research Infrastructure (PHIRI) project (available from: https://cordis.europa.eu/project/id/101018317/fr). PHIRI is funded by the European Union's Horizon 2020 research and innovation program under grant agreement No: 101018317.

The protocol was developed in collaboration with all listed institutions. The funding body was not involved in the design of the study; it will not be involved in the collection, analysis and interpretation of data or in writing the manuscript. The content of this publication will represent the views of the authors only and not of the European Commission or any other body of the European Union. The European Commission does not take any responsibility for use that may be made of the information it will contain.

Competing interests statement

Authors declare no competing interests.

For peer review only

REFERENCES

1. Cucinotta D, Vanelli M. WHO Declares COVID-19 a Pandemic. Acta Biomed. 2020;91(1):157-60.
2. Novel Coronavirus (2019-nCoV) Situation Report – 22 (World Health Organization). 2020 [Available from: https://www.who.int/docs/default-source/coronaviruse/situation-reports/20200211-sitrep-22-ncov.pdf.
3. World Health Organisation (WHO). [Available from: https://www.who.int/emergencies/diseases/novel-coronavirus-2019/situation-reports.
4. Cheng Y, Luo R, Wang K, Zhang M, Wang Z, Dong L, et al. Kidney disease is associated with in-hospital death of patients with COVID-19. Kidney Int. 2020;97(5):829-38.
5. Kumar A, Arora A, Sharma P, Anikhindi SA, Bansal N, Singla V, et al. Is diabetes mellitus associated with mortality and severity of COVID-19? A meta-analysis. Diabetes Metab Syndr. 2020;14(4):535-45.
6. Grasselli G, Greco M, Zanella A, Albano G, Antonelli M, Bellani G, et al. Risk Factors Associated With Mortality Among Patients With COVID-19 in Intensive Care Units in Lombardy, Italy. JAMA Intern Med. 2020;180(10):1345-55.
7. Levin AT, Hanage WP, Owusu-Boaitey N, Cochran KB, Walsh SP, Meyerowitz-Katz G. Assessing the age specificity of infection fatality rates for COVID-19: systematic review, meta-analysis, and public policy implications. European Journal of Epidemiology. 2020;35(12):1123-38.
8. Himmels JPW, Borge TC, Brurberg KG, Gravningen KM. COVID-19: COVID-19 and risk factors for hospital admission, severe disease and death Oslo: Norwegian Institute of Public Health; 2021.
9. Johns Hopkins University of Medicine; Coronavirus resource centre. COVID-19 Dashboard by the Center for Systems Science and Engineering (CSSE) at Johns Hopkins University (JHU) [Available from: https://coronavirus.jhu.edu/map.html.
10. Andrews N, Stowe J, Kirsebom F, Toffa S, Rickeard T, Gallagher E, et al. Effectiveness of COVID-19 vaccines against the Omicron (B.1.1.529) variant of concern. medRxiv. 2021:2021.12.14.21267615.
11. Clegg A, Young J, Iliffe S, Rikkert MO, Rockwood K. Frailty in elderly people. Lancet. 2013;381(9868):752-62.
12. van den Akker M, Buntinx F, Knottnerus JA. Comorbidity or multimorbidity: what's in a name? A review of literature. European Journal of General Practice. 1996;2(2):65-70.
13. Fried LP, Tangen CM, Walston J, Newman AB, Hirsch C, Gottdiener J, et al. Frailty in older adults: evidence for a phenotype. J Gerontol A Biol Sci Med Sci. 2001;56(3):M146-56.
14. Rockwood K, Song X, MacKnight C, Bergman H, Hogan DB, McDowell I, et al. A global clinical measure of fitness and frailty in elderly people. Cmaj. 2005;173(5):489-95.
15. Coste J, Valderas JM, Carcaillon-Bentata L. Estimating and characterizing the burden of multimorbidity in the community: A comprehensive multistep analysis of two large nationwide representative surveys in France. PLoS Med. 2021;18(4):e1003584.
16. Iaccarino G, Grassi G, Borghi C, Ferri C, Salvetti M, Volpe M. Age and Multimorbidity Predict Death Among COVID-19 Patients: Results of the SARS-RAS Study of the Italian Society of Hypertension. Hypertension. 2020;76(2):366-72.
17. McQueenie R, Foster HME, Jani BD, Katikireddi SV, Sattar N, Pell JP, et al. Multimorbidity, polypharmacy, and COVID-19 infection within the UK Biobank cohort. PLoS One. 2020;15(8):e0238091.
18. Woolford SJ, D'Angelo S, Curtis EM, Parsons CM, Ward KA, Dennison EM, et al. COVID-19 and associations with frailty and multimorbidity: a prospective analysis of UK Biobank participants. Aging Clin Exp Res. 2020;32(9):1897-905.
19. Zhang XM, Jiao J, Cao J, Huo XP, Zhu C, Wu XJ, et al. Frailty as a predictor of mortality among patients with COVID-19: a systematic review and meta-analysis. BMC Geriatr. 2021;21(1):186.
20. Yang Y, Luo K, Jiang Y, Yu Q, Huang X, Wang J, et al. The Impact of Frailty on COVID-19 Outcomes: A Systematic Review and Meta-analysis of 16 Cohort Studies. J Nutr Health Aging. 2021;25(5):702-9.

For peer review only - http://bmjopen.bmj.com/site/about/guidelines.xhtml

21.Saragih ID, Advani S, Saragih IS, Suarilah I, Susanto I, Lin CJ. Frailty as a mortality predictor in older adults with COVID-19: A systematic review and meta-analysis of cohort studies. Geriatr Nurs. 2021;42(5):983-92.
22.Pranata R, Henrina J, Lim MA, Lawrensia S, Yonas E, Vania R, et al. Clinical frailty scale and mortality in COVID-19: A systematic review and dose-response meta-analysis. Arch Gerontol Geriatr. 2021;93:104324.
23.Maltese G, Corsonello A, Di Rosa M, Soraci L, Vitale C, Corica F, et al. Frailty and COVID-19: A Systematic Scoping Review. J Clin Med. 2020;9(7).
24.Kastora S, Kounidas G, Perrott S, Carter B, Hewitt J, Myint PK. Clinical frailty scale as a point of care prognostic indicator of mortality in COVID-19: a systematic review and meta-analysis. EClinicalMedicine. 2021;36:100896.
25.Cosco TD, Best J, Davis D, Bryden D, Arkill S, van Oppen J, et al. What is the relationship between validated frailty scores and mortality for adults with COVID-19 in acute hospital care? A systematic review. Age Ageing. 2021;50(3):608-16.
26.Dumitrascu F, Branje KE, Hladkowicz ES, Lalu M, McIsaac DI. Association of frailty with outcomes in individuals with COVID-19: A living review and meta-analysis. Journal of the American Geriatrics Society. 2021;69(9):2419-29.
27.Mair FS, Foster HM, Nicholl BI. Multimorbidity and the COVID-19 pandemic - An urgent call to action. J Comorb. 2020;10:2235042x20961676.
28.Mamelund S-E, Shelley-Egan C, Rogeberg O. The association between socioeconomic status and pandemic influenza: Systematic review and meta-analysis. PloS one. 2021;16(9):e0244346.
29.Niedzwiedz CL, O'Donnell CA, Jani BD, Demou E, Ho FK, Celis-Morales C, et al. Ethnic and socioeconomic differences in SARS-CoV-2 infection: prospective cohort study using UK Biobank. BMC medicine. 2020;18:1-14.
30.Nash D, Qasmieh S, Robertson M, Rane M, Zimba R, Kulkarni S, et al. Household factors and the risk of severe COVID-like illness early in the US pandemic. medRxiv. 2020.
31.Himmels JPW, Qureshi SA, Brurberg KG, Gravningen KM. COVID-19: Long-Term Effects of COVID-19. Rapid review. Oslo: Norwegian Institute of Public Health, 2021. 2021.
32.Dumitrascu F, Branje KE, Hladkowicz ES, Lalu M, McIsaac DI. Association of frailty with outcomes in individuals with COVID-19: A living review and meta-analysis. J Am Geriatr Soc. 2021;69(9):2419-29.
33.National Institute for Health Research. PROSPERO; International prospective register of systematic reviews [Available from: https://www.crd.york.ac.uk/PROSPERO/.
34.Shamseer L, Moher D, Clarke M, Ghersi D, Liberati A, Petticrew M, et al. Preferred reporting items for systematic review and meta-analysis protocols (PRISMA-P) 2015: elaboration and explanation. Bmj. 2015;350:g7647.
35.Morgan RL, Whaley P, Thayer KA, Schünemann HJ. Identifying the PECO: A framework for formulating good questions to explore the association of environmental and other exposures with health outcomes. Environ Int. 2018;121(Pt 1):1027-31.
36.Morgan RL, Thayer KA, Bero L, Bruce N, Falck-Ytter Y, Ghersi D, et al. GRADE: Assessing the quality of evidence in environmental and occupational health. Environ Int. 2016;92-93:611-6.
37.Almirall J, Fortin M. The coexistence of terms to describe the presence of multiple concurrent diseases. J Comorb. 2013;3:4-9.
38.Johnston MC, Crilly M, Black C, Prescott GJ, Mercer SW. Defining and measuring multimorbidity: a systematic review of systematic reviews. Eur J Public Health. 2019;29(1):182-9.
39.St Sauver JL, Boyd CM, Grossardt BR, Bobo WV, Rutten LJF, Roger VL, et al. Risk of developing multimorbidity across all ages in an historical cohort study: differences by sex and ethnicity. BMJ open. 2015;5(2):e006413.
40.Gail MH. Population based study. In: Armitage, &, Colton, editors. Encyclopedia of biostatistics: Wiley, Chichester 1998. p. 3450.
41.Lieb R. Population-Based Study. In: Gellman MD, Turner JR, editors. Encyclopedia of Behavioral Medicine. New York, NY: Springer New York; 2013. p. 1507-8.

For peer review only - http://bmjopen.bmj.com/site/about/guidelines.xhtml

42. World Health Organisation (WHO). COVID-19 Global literature on coronavirus disease 2022 [Available from: https://search.bvsalud.org/global-literature-on-novel-coronavirus-2019-ncov/.
43. Lazarus JV, Palayew A, Rasmussen LN, Andersen TH, Nicholson J, Norgaard O. Searching PubMed to Retrieve Publications on the COVID-19 Pandemic: Comparative Analysis of Search Strings. J Med Internet Res. 2020;22(11):e23449.
44. Makovski TT, Schmitz S, Zeegers MP, Stranges S, van den Akker M. Multimorbidity and quality of life: Systematic literature review and meta-analysis. Ageing Res Rev. 2019;53:100903.
45. Ouzzani M, Hammady H, Fedorowicz Z, Elmagarmid A. Rayyan-a web and mobile app for systematic reviews. Syst Rev. 2016;5(1):210.
46. Page MJ, Moher D, Bossuyt PM, Boutron I, Hoffmann TC, Mulrow CD, et al. PRISMA 2020 explanation and elaboration: updated guidance and exemplars for reporting systematic reviews. Bmj. 2021;372:n160.
47. Alderson P, Green S, Higgins JPT, editors. Cochrane Reviewers' Handbook 4.2.2 [updated March 2004]. In: The Cochrane Library, Issue 1, Chichester, UK: John Wiley & Sons, Ltd. 2004. .
48. Centre for Reviews and Dissemination [CRD]. Systematic Reviews: CRD's Guidance for Undertaking Reviews in Healthcare. 3rd edn. . CRD University of York, York Publishing Services Ltd, York, . 2009.
49. Wells GA, Shea B, O'Connell D, Peterson J, Welch V, Losos M, et al. The Newcastle-Ottawa Scale (NOS) for assessing the quality if nonrandomized studies in meta-analyses. Available from: http://www.ohri.ca/programs/clinical_epidemiology/oxford.asp [
50. Herzog R, Álvarez-Pasquin MJ, Díaz C, Del Barrio JL, Estrada JM, Gil Á. Are healthcare workers' intentions to vaccinate related to their knowledge, beliefs and attitudes? a systematic review. BMC Public Health. 2013;13(1):154.
51. Modesti PA, Reboldi G, Cappuccio FP, Agyemang C, Remuzzi G, Rapi S, et al. Panethnic differences in blood pressure in Europe: a systematic review and meta-analysis. PloS one. 2016;11(1):e0147601.
52. Page MJ MJ, Bossuyt PM, Boutron I, Hoffmann TC, Mulrow CD, et al. . The PRISMA 2020 statement: an updated guideline for reporting systematic reviews. . BMJ 2021:372:n71. .
53. Schisterman EF, Cole SR, Platt RW. Overadjustment bias and unnecessary adjustment in epidemiologic studies. Epidemiology. 2009;20(4):488-95.
54. Viechtbauer W. Conducting Meta-Analyses in R with the metafor Package. Journal of Statistical Software. 2010;36(3):1 - 48.
55. Begg CB, Mazumdar M. Operating Characteristics of a Rank Correlation Test for Publication Bias. Biometrics. 1994;50(4):1088-101.
56. Egger M, Davey Smith G, Schneider M, Minder C. Bias in meta-analysis detected by a simple, graphical test. Bmj. 1997;315(7109):629-34.
57. Duval S, Tweedie R. Trim and fill: A simple funnel-plot-based method of testing and adjusting for publication bias in meta-analysis. Biometrics. 2000;56(2):455-63.
58. Higgins JP, Thompson SG. Quantifying heterogeneity in a meta-analysis. Stat Med. 2002;21(11):1539-58.
59. Tuty Kuswardhani RA, Henrina J, Pranata R, Anthonius Lim M, Lawrensia S, Suastika K. Charlson comorbidity index and a composite of poor outcomes in COVID-19 patients: A systematic review and meta-analysis. Diabetes & Metabolic Syndrome: Clinical Research & Reviews. 2020;14(6):2103-9.
60. Vist GE, Arentz-Hansen EH, Vedøy TF, Spilker RACS, Hafstad EV, Giske L. Incidence and severe outcomes from COVID-19 among immigrant and minority ethnic groups and among groups of different socio-economic status: A systematic review. 2021.
61. Wachtler B, Michalski N, Nowossadeck E, Diercke M, Wahrendorf M, Santos-Hövener C, et al. Socioeconomic inequalities and COVID-19–A review of the current international literature. 2020.
62. Khanijahani A, Iezadi S, Gholipour K, Azami-Aghdash S, Naghibi D. A systematic review of racial/ethnic and socioeconomic disparities in COVID-19. International journal for equity in health. 2021;20(1):1-30.

For peer review only

63. Lewin S, Glenton C, Munthe-Kaas H, Carlsen B, Colvin CJ, Gülmezoglu M, et al. Using qualitative evidence in decision making for health and social interventions: an approach to assess confidence in findings from qualitative evidence syntheses (GRADE-CERQual). PLoS Med. 2015;12(10):e1001895.
64. Tong A, Flemming K, McInnes E, Oliver S, Craig J. Enhancing transparency in reporting the synthesis of qualitative research: ENTREQ. BMC medical research methodology. 2012;12(1):1-8.
65. WHO Regional Office for Europe. Guide to qualitative evidence synthesis: evidence-informed policy-making using research in the EVIPNET framework. 2021.

For peer review only

For peer review only - http://bmjopen.bmj.com/site/about/guidelines.xhtml

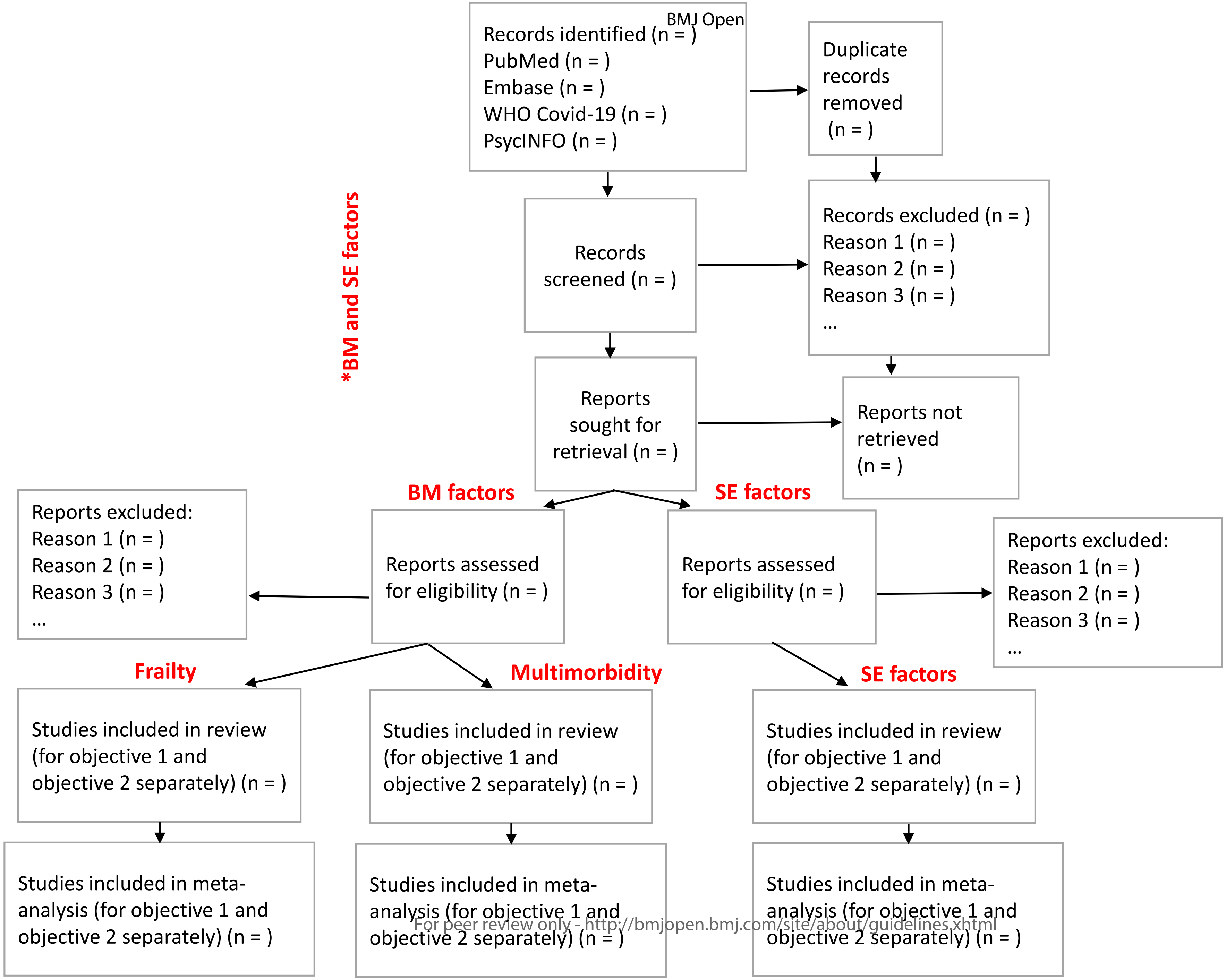

For peer review only - http://bmjopen.bmj.com/site/about/guidelines.xhtml

WHO COVID-19 Global literature on coronavirus disease strategy

((tw:("socio economic" OR "socio-economic" OR socioeconomics OR "socio-economics" OR "socio economics" OR socioeconomic OR "social difference" OR "social differences" OR "social inequality" OR "social inequalities" OR "socioeconomic inequality" OR "socioeconomic inequalities" OR "social disparity" OR "social disparities" OR education OR literacy OR "socioprofessional" OR "socio-professional" OR "socio professional" OR "social conditions" OR "social class" OR "social classes" OR "health status disparity" OR "health status disparities" OR income OR poverty OR deprivation OR rural OR urban OR "housing deprivation" OR homeless OR houseless OR homelessness OR ethnic$ OR race OR emigrant OR immigrant OR migrant OR "minority group" OR "minority groups" OR disadvantaged OR "marital status" OR ((characteristics OR factors OR status) AND (economic OR social OR educational)) OR ((composition OR characteristics OR size) AND (family OR household)))) OR (tw:(multimorbidity OR "multi-morbidity" OR "multi morbidity" OR multimorbidities OR "multi-morbidities" OR "multi morbidities" OR multimorbid OR "multi-morbid" OR "multi morbid" OR comorbidity OR "co-morbidity" OR "co morbidity" OR comorbidities OR "co-morbidities" OR "co morbidities" OR comorbid OR "co morbid" OR "multiple chronic conditions" OR "multiple chronic diseases" OR "multiple conditions" OR "multiple diseases" OR "multiple disorders" OR polymorbid$ OR "poly-morbidity" OR "poly-morbidities" OR "poly morbidity" OR "poly morbidities" OR polypath$ OR pluripath$ OR multipath$ OR "multi pathology" OR "multi pathologies" OR "multi-pathology" OR "multi-pathologies" OR "multiple pathologies" OR "disease cluster" OR "disease clusters" OR "disease pattern" OR "disease patterns" OR "concurrent chronic diseases" OR "multiple chronic disorders")) OR (tw:(frailty OR frail))) AND tw:("cross-sectional" OR "cross sectional" OR "case-control" OR "case control" OR cohort OR longitudinal OR "ecological study" OR "ecological studies" OR "ecological design" OR "ecological designs" OR observational OR "observational study" OR "observational studies" OR "observational design" OR "observational designs" OR "prospective study" OR "prospective studies" OR "prospective design" OR "prospective designs" OR "retrospective study" OR "retrospective studies" OR "retrospective design" OR "retrospective designs" OR "prospective observational study" OR "prospective observational studies" OR "retrospective observational study" OR "retrospective observational studies")

For peer review only

For peer review only - http://bmjopen.bmj.com/site/about/guidelines.xhtml

PsycINFO strategy

S1

"cross-sectional" OR "cross sectional" OR "case-control" OR "case control" OR cohort OR longitudinal OR "ecological study" OR "ecological studies" OR "ecological design" OR "ecological designs" OR observational OR "observational study" OR "observational studies" OR "observational design" OR "observational designs" OR "prospective study" OR "prospective studies" OR "prospective design" OR "prospective designs" OR "retrospective study" OR "retrospective studies" OR "retrospective design" OR "retrospective designs" OR "prospective observational study" OR "prospective observational studies" OR "retrospective observational study" OR "retrospective observational studies" OR DE("Longitudinal Studies" OR "Followup studies" OR "Retrospective studies" OR "Cohort analysis")

S2

"socio economic" OR "socio-economic" OR socioeconomics OR "socio-economics" OR "socio economics" OR socioeconomic OR "social difference" OR "social differences" OR "social inequality" OR "social inequalities" OR "socioeconomic inequality" OR "socioeconomic inequalities" OR "social disparity" OR "social disparities" OR education OR literacy OR "socioprofessional" OR "socio-professional" OR "socio professional" OR "social conditions" OR "social class" OR "social classes" OR "health status disparity" OR "health status disparities" OR income OR poverty OR deprivation OR rural OR urban OR "housing deprivation" OR homeless OR houseless OR homelessness OR ethnic* OR race OR emigrant OR immigrant OR migrant OR "minority group" OR "minority groups" OR disadvantaged OR "marital status" OR ((characteristics OR factors OR status) AND (economic OR social OR educational)) OR ((composition OR characteristics OR size) AND (family OR household)) OR DE ("Disadvantaged" OR "Economic Disadvantage" OR "Economic Inequality" OR "Economic Resources" OR "Family Socioeconomic Level" OR "Income (Economic)" OR "Lower Class" OR "Poverty" OR "Rural Health" OR "Socioeconomic Status" OR "Socioeconomic Factors" OR "Social Class" OR "Urban Health" OR "Racial and Ethnic Differences" OR "Racial Disparities" OR "Health Disparities")

S3

multimorbidity OR "multi-morbidity" OR "multi morbidity" OR multimorbidities OR "multi-morbidities" OR "multi morbidities" OR multimorbid OR "multi-morbid" OR "multi morbid" OR comorbidity OR "co-morbidity" OR "co morbidity" OR comorbidities OR "co-morbidities" OR "co morbidities" OR comorbid OR "co morbid" OR "multiple chronic conditions" OR "multiple chronic diseases" OR "multiple conditions" OR "multiple diseases" OR "multiple disorders" OR polymorbid* OR "poly-morbid" OR "poly-morbidity" OR "poly-morbidities" OR "poly morbid" OR "poly morbidity" OR "poly morbidities" OR polypath* OR pluripath* OR multipath* OR "multi pathology" OR "multi pathologies" OR "multi-pathology" OR "multi-pathologies" OR "multiple pathologies" OR "disease cluster" OR "disease clusters" OR "disease pattern" OR "disease patterns" OR "concurrent chronic diseases" OR "multiple chronic disorders" OR Comorbidity [DE]

S4 Frailty OR frail

S5

"covid-19" OR "2019-ncov" OR "sars cov 2" OR "cov-19" OR cov19 OR "cov 19" OR 2019nCoV OR betacoronavirus* OR "corona virus" OR coronavirus* OR coronovirus* OR CoV OR CoV2 OR COVID OR covid19 OR "covid 2019" OR "Severe Acute Respiratory Syndrome Coronavirus 2" OR "2019 ncov" OR (wuhan AND Coronavirus) OR "HCoV-19" OR nCoV OR SARS2 OR SARSCoV OR (sars AND virus) OR "sars virus" OR (sars AND CoV) OR "sars cov" OR "severe acute respiratory syndrome cov" OR "SARS CoV-2" OR "SARS-

For peer review only

PsycINFO strategy

CoV-2" OR SARSCoV2 OR "CoV-2" OR "covid 19" OR covid2019 OR "covid-2019" OR "novel CoV" OR "corona pandemic" OR "wuhan virus" OR "CoV 2" OR ((wuhan OR hubei OR huanan) AND ("severe acute respiratory" OR pneumonia*) AND outbreak*) OR COVID-19 [DE]

S6

(S2 OR S3 OR S4) AND S5 AND S1

For peer review only

Embase strategy

((comorbid* OR 'comorbidity'/exp OR comorbidity OR 'multiple chronic conditions'/exp OR 'multiple chronic conditions' OR multimorbid* OR 'multi morbid' OR 'multi morbidity' OR 'multi morbidities' OR 'co morbid' OR 'co morbidity' OR 'co morbidities' OR 'disease pattern' OR 'disease patterns' OR 'disease cluster' OR 'disease clusters' OR 'multiple conditions' OR 'multiple diseases' OR 'multiple disorders' OR polymorbid* OR 'poly morbid' OR 'poly morbidity' OR 'poly morbidities' OR polypath* OR 'multiple pathology' OR 'multiple pathologies') OR ('frail elderly'/exp OR 'frail elderly' OR 'frailty'/exp OR 'frailty' OR frail*) OR (('socioeconomics'/exp OR 'socioeconomics' OR 'social status'/exp OR 'social status' OR 'social stratification'/exp OR 'social stratification' OR 'social class'/exp OR 'social class') OR ('urban area'/exp OR 'urban area' OR 'rural area'/exp OR 'rural area') OR 'population group'/exp OR ('minority group'/exp OR 'minority group' OR 'homelessness'/exp OR 'homelessness' OR 'migrant'/exp OR 'migrant' OR 'race'/exp OR 'race' OR ethnic* OR 'minority groups') OR ('economic status'/exp OR 'economic status' OR 'income'/exp OR 'income' OR 'income group'/exp OR 'income group') OR ('social discrimination'/exp OR 'social discrimination' OR 'socioeconomic inequality'/exp OR 'socioeconomic inequality' OR 'health disparity'/exp OR 'health disparity' OR 'poverty'/exp OR 'poverty' OR 'income inequality'/exp OR 'income inequality') OR ('educational status'/exp OR 'educational status' OR 'literacy'/exp OR 'literacy' OR 'education* level') OR ('family size'/exp OR 'family size' OR 'household characteristics' OR 'household size' OR 'household'/exp))) AND ('longitudinal study'/exp OR 'longitudinal study' OR 'cross-sectional study'/exp OR 'cross-sectional study' OR longitudinal OR 'cross sectional' OR 'case control study'/exp OR 'case control study' OR 'case control' OR cohort OR 'ecological study' OR 'ecological design' OR 'observational study'/exp OR 'observational study' OR 'retrospective study'/exp OR 'retrospective study' OR 'prospective study'/exp OR 'prospective study' OR 'prospective observational study' OR 'prospective observational studies' OR 'retrospective observational study' OR 'retrospective observational studies' OR 'ecological designs' OR 'prospective studies' OR 'retrospective studies' OR 'observational studies' OR 'ecological studies') AND ('coronavirus disease 2019'/exp OR 'coronavirus disease 2019' OR 'covid 2019' OR 'covid 19' OR covid2019 OR covid19 OR sarscov2 OR 'sars cov 2' OR 'sars cov2' OR 'severe acute respiratory syndrome'/exp OR 'severe acute respiratory syndrome' OR 'wuhan virus' OR 'cov 2' OR cov2 OR coronavirus OR 'corona virus' OR 'novel cov' OR 2019ncov OR '2019 ncov' OR 'corona pandemic' OR betacoronavirus OR cov19 OR 'cov 19' OR 'hcov-19' OR ncov OR sars2) AND [english]/lim AND [2020-2021]/py

For peer review only

PRISMA-P (Preferred Reporting Items for Systematic review and Meta-Analysis Protocols) 2015 checklist: recommended items to address in a systematic review protocol*

Section and topic	Item No	Checklist item	
**ADMINISTRATIVE INFORMATION**			
Title:			
Identification	1a	Identify the report as a protocol of a systematic review	1a
Update	1b	If the protocol is for an update of a previous systematic review, identify as such	
Registration	2	If registered, provide the name of the registry (such as PROSPERO) and registration number	Page 5, line 151
Authors:			
Contact	3a	Provide name, institutional affiliation, e-mail address of all protocol authors; provide physical mailing address of corresponding author	Page 1
Contributions	3b	Describe contributions of protocol authors and identify the guarantor of the review	Page 14, lines 404-412
Amendments	4	If the protocol represents an amendment of a previously completed or published protocol, identify as such and list changes; otherwise, state plan for documenting important protocol amendments	Page 5, lines 152,153
Support:			
Sources	5a	Indicate sources of financial or other support for the review	Page 14, 414-421
Sponsor	5b	Provide name for the review funder and/or sponsor	Page 14, 414-421
Role of sponsor or funder	5c	Describe roles of funder(s), sponsor(s), and/or institution(s), if any, in developing the protocol	Page 14, 414-421
**INTRODUCTION**			
Rationale	6	Describe the rationale for the review in the context of what is already known	Page 4, 5, lines 86-129
Objectives	7	Provide an explicit statement of the question(s) the review will address with reference to participants, interventions, comparators, and outcomes (PICO)	Page 5, lines 132-146 and Page 6, lines 160-172
**METHODS**			
Eligibility criteria	8	Specify the study characteristics (such as PICO, study design, setting, time frame) and report characteristics (such as years considered, language, publication status) to be used as criteria for eligibility for the review	Page 6, lines 160-172 and Page 9, lines 240-269
Information sources	9	Describe all intended information sources (such as electronic databases, contact with study authors, trial registers or other grey literature sources) with planned dates of coverage	Page 8 and 9, lines 209-211,

For peer review only

			217-220
Search strategy	10	Present draft of search strategy to be used for at least one electronic database, including planned limits, such that it could be repeated	Page 7 and 8
Study records:			
Data management	11a	Describe the mechanism(s) that will be used to manage records and data throughout the review	Page 9, lines 224-233
Selection process	11b	State the process that will be used for selecting studies (such as two independent reviewers) through each phase of the review (that is, screening, eligibility and inclusion in meta-analysis)	Page 9 and 10, lines 236-277
Data collection process	11c	Describe planned method of extracting data from reports (such as piloting forms, done independently, in duplicate), any processes for obtaining and confirming data from investigators	Page 10, lines 279-283
Data items	12	List and define all variables for which data will be sought (such as PICO items, funding sources), any pre-planned data assumptions and simplifications	Page 10, lines 279-283
Outcomes and prioritization	13	List and define all outcomes for which data will be sought, including prioritization of main and additional outcomes, with rationale	Page 6, lines 163,164, 171,172
Risk of bias in individual studies	14	Describe anticipated methods for assessing risk of bias of individual studies, including whether this will be done at the outcome or study level, or both; state how this information will be used in data synthesis	Page 10 and 11, lines 285-299
Data synthesis	15a	Describe criteria under which study data will be quantitatively synthesised	Page 11, lines 301-320
	15b	If data are appropriate for quantitative synthesis, describe planned summary measures, methods of handling data and methods of combining data from studies, including any planned exploration of consistency (such as I^2, Kendall's τ)	Page 11, lines 319-324
	15c	Describe any proposed additional analyses (such as sensitivity or subgroup analyses, meta-regression)	Page 11, lines 324-327
	15d	If quantitative synthesis is not appropriate, describe the type of summary planned	Page 11, lines 309-311
Meta-bias(es)	16	Specify any planned assessment of meta-bias(es) (such as publication bias across studies, selective reporting within studies)	Page 11, 321-323
Confidence in cumulative evidence	17	Describe how the strength of the body of evidence will be assessed (such as GRADE)	GRADE assessment is not foreseen at this stage; only the individual study quality assessment is planned using Newcastle-

For peer review only

	Ottawa scales

*** It is strongly recommended that this checklist be read in conjunction with the PRISMA-P Explanation and Elaboration (cite when available) for important clarification on the items. Amendments to a review protocol should be tracked and dated. The copyright for PRISMA-P (including checklist) is held by the PRISMA-P Group and is distributed under a Creative Commons Attribution Licence 4.0.**

From: Shamseer L, Moher D, Clarke M, Ghersi D, Liberati A, Petticrew M, Shekelle P, Stewart L, PRISMA-P Group. Preferred reporting items for systematic review and meta-analysis protocols (PRISMA-P) 2015: elaboration and explanation. BMJ. 2015 Jan 2;349(jan02 1):g7647.

For peer review only
